# Increased flushing frequency of a model plumbing system initially promoted the formation of viable but non culturable cells but ultimately reduced the concentration of culturable and total *Legionella* DNA

**DOI:** 10.1016/j.heliyon.2024.e32334

**Published:** 2024-06-04

**Authors:** Muhammad Atif Nisar, Kirstin E. Ross, Melissa H. Brown, Richard Bentham, Giles Best, Nicholas S. Eyre, Sophie C. Leterme, Harriet Whiley

**Affiliations:** a College of Science and Engineering, Flinders University, Bedford Park, SA, 5042, Australia; b ARC Training Centre for Biofilm Research and Innovation, Flinders University, Bedford Park, SA, 5042, Australia; c College of Medicine and Public Health, Flinders University, Bedford Park, SA, 5042, Australia; d Flow Cytometry Facility, Flinders University, Bedford Park, SA, 5042, Australia; e Institute for Nanoscience and Technology, Flinders University, Bedford Park, SA, 5042, Australia

**Keywords:** Legionnaires' disease, Viable but non culturable (VBNC) *Legionella*, Building plumbing system, Water stagnation, Flow dynamics, Free-living amoebae

## Abstract

*Legionella* is the causative agent of Legionnaires’ disease, and its prevalence in potable water is a significant public health issue. Water stagnation within buildings increases the risk of *Legionella.* However, there are limited studies investigating how stagnation arising through intermittent usage affects *Legionella* proliferation and the studies that are available do not consider viable but non culturable (VBNC) *Legionella*. This study used a model plumbing system to examine how intermittent water stagnation affects both VBNC and culturable *Legionella*. The model plumbing system contained a water tank supplying two biofilm reactors. The model was initially left stagnant for ≈5 months (147 days), after which one reactor was flushed daily, and the other weekly. Biofilm coupons, and water samples were collected for analysis at days 0, 14 and 28. These samples were analysed for culturable and VBNC *Legionella*, free-living amoebae, and heterotrophic bacteria. After 28 days, once-a-day flushing significantly (*p* < 0.001) reduced the amount of biofilm-associated culturable *Legionella* (1.5 log_10_ reduction) compared with weekly flushing. However, higher counts of biofilm-associated VBNC *Legionella* (1 log_10_ higher) were recovered from the reactor with once-a-day flushing compared with weekly flushing. Likewise, once-a-day flushing increased the population of biofilm-associated *Vermamoeba vermiformis* (approximately 3 log_10_ higher) compared with weekly flushing, which indicated a positive relationship between VBNC *Legionella* and *V. vermiformis*. This is the first study to investigate the influence of stagnation on VBNC *Legionella* under environmental conditions. Overall, this study showed that a reduction in water stagnation decreased culturable *Legionella* but not VBNC *Legionella*.

## Highlights

• Once-a-day flushing reduced culturable *Legionella* compared with weekly flushing.
• Increased water flushing promoted formation of viable but non culturable *Legionella.*
• Heterotrophic bacteria and amoebae were positively associated with *Legionella* growth.

## 1 Introduction

Opportunistic premise plumbing pathogens (OPPP) are environmental waterborne pathogens that opportunistically cause disease in vulnerable individuals [1]. *Legionella pneumophila* is OPPP associated with hospital and community acquired infections. It is the causative agent of Pontiac Fever, an acute “flu-like” illness and Legionnaires' Disease, a severe atypical pneumonia like infection [2]. The genus *Legionella* consists of more than 60 species and 80 distinct serogroups (sg) [3], with *L*. *pneumophila* sg1 being the primary etiological agent of Legionnaires' Disease and responsible for 70 %–92 % of reported cases [4]. Globally, the incidence of legionellosis has been increasing [5]. In 2022, the European Legionnaires' Disease Surveillance Network documented 8372 confirmed cases of Legionnaires’ Disease, of which 5.1 % were hospital acquired and 66.9 % were community acquired infections [6].

*Legionella* is ubiquitous in natural and manufactured water bodies. Cooling towers, humidifiers, engineered water systems, recreational water, and building plumbing systems are major reservoirs of *Legionella* [2]. Intrinsic resistance against commercially available disinfectants, mutualistic and symbiotic relationships with protozoans, and growth within multispecies biofilms, are key biotic factors responsible for the survival and persistence of *Legionella* in manufactured water systems [1,7,8]. *Legionella* spp. are intracellular parasites of various freshwater protozoan species, such as amoebae (*Acanthamoeba*, *Naegleria*, *Vahlkampfia*, and *Vermamoeba vermiformis* (previously known as *Hartmannella vermiformis)*) and ciliates (*Paramecium* and *Tetrahymena*) [9]. Members of gymnamoebae, noticeably *V. vermiformis* and *Acanthamoeba*, have been identified as major reservoirs and vehicles of *Legionella* in both hospital and domestic water systems [[10], [11], [12]]. Amoebae trophozoites support the intracellular division and biogenesis of potentially infectious and highly pathogenic viable but nonculturable (VBNC) *Legionella.* These amoebic cysts have been demonstrated to protect *Legionella* from prolonged disinfection treatments [10,13].

Abiotic factors such as hydraulic dynamics, age, plumbing system materials, water stagnation, corrosion, water temperature and inadequate disinfection procedures contribute to the growth and persistence of *Legionella* in engineered water systems [2,14]. In building plumbing systems, aerators, balancing valves, dead ends, dead legs, diffusers, flow restrictors, and intermitted usage all impact hydraulic dynamics and promote temporary or permanent water stagnation [15]. This is often exacerbated in green building plumbing systems that have been designed deliberately to reduce water usage [16,17]. Recently, during the COVID-19 pandemic, prolonged periods of lockdown caused extreme water stagnation in complicated plumbing systems of commercial buildings [18]. Both inappropriate hydraulic dynamics and water stagnation are likely to result in the failure of disinfection treatments, corrosion, and accumulation of sediments and nutrients. Previous studies, as well as government regulations from across the globe, recommend the elimination of factors promoting water stagnation within engineered water systems to minimize the risk of *Legionella* [15,19,20]. However, this has been contradicted by several studies that suggest that avoiding conditions promoting water stagnation has no effect on *Legionella* persistence. It has been proposed that water stagnation restricts the delivery of nutrients, whereas recirculation of water provides nutrients to the point of delivery (e.g., taps, showers, etc.) ultimately promoting OPPP regrowth in building plumbing system [21,22]. Many biofilm studies have identified that flow of the bathing medium promotes more robust biofilm formation and attachment [22]. Due to the complex relationship between different biotic and abiotic factors, it is always challenging to study the role of water stagnation on the survival and persistence of *Legionella* and associated host amoebae in engineered water systems. This is further complicated by the complexities and sensitivities of different *Legionella* detection methods. In engineered water systems, bacteriological culturing and quantitative PCR (qPCR) are the standard methods used to detect *Legionella* contamination [23,24]; however, neither assay provides any valuable information about VBNC *Legionella*. Most previous studies conducted to examine the relationship between *Legionella* and water stagnation have focused on culturable *Legionella* [19,21,22]. However, quantification of both culturable and VBNC states is necessary to monitor the survival of viable and potentially infectious *Legionella* populations. This study used a model plumbing system to investigate the effect of water stagnation and hydraulic dynamics on the survival and persistence of VBNC and culturable *Legionella* in engineered water systems.

## 2 Materials and methods

This study used a model plumbing system with two bioreactors that was left stagnant for 147 days, after which one reactor was flushed with approximately 70 L water once-a-day (high usage), and the other once-a-week (low usage i.e., temporary stagnation). VBNC *Legionella* were enumerated using a newly described technique [25], which was conducted concurrently with traditional culture and qPCR methods. The amoebic hosts *Acanthamoeba* and *V. vermiformis* were also enumerated via culture and qPCR and total bacteria were via a heterotrophic plate count. The effect of changing hydraulic conditions on biofilm communities, and the relationship between *Legionella* and amoeba hosts, was also examined using fluorescent *in situ* hybridization and confocal scanning microscopy.

### 2.1 Model plumbing system

**Fig. 1:**
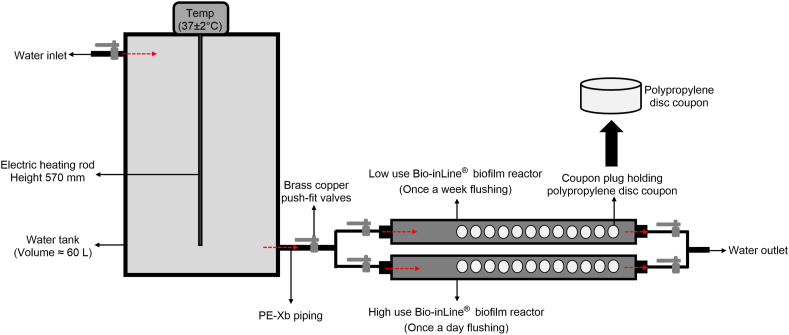
Overview of model plumbing system constructed to examine the effect flushing frequency on *Legionella* persistence. Red arrow indicates unidirectional flow of water.

A simulated building plumbing system was constructed (Fig. 1). It consisted of a 60 L capacity plastic water tank (DR5060, AdMerch) connected to a tap that received municipal potable water and two Bio-inLine® biofilm reactors (IBR 500, BioSurface Technologies Corporation). Each IBR contained 12 polypropylene disc coupons (diameter = 12.7 mm; RD128-PP, BioSurface Technologies Corporation). To maintain the water temperature at 37 ± 2 °C, a 2 m long immersion electric heater with a digital thermostat (SKU BDIH2400W, Scintex®) was fitted in the water tank and used to measured water temperature. This optimum growth temperature of *Legionella* was selected to represent the worst-case scenario within a building plumbing system. The IBRs were connected via PE-Xb piping (4950091, SmarteX™) and brass copper push-fit connectors (elbow: 4700328, slip-coupling: 4700354 and tee: 4700360, SmarteX™). To control unidirectional water flow, brass copper push-fit valves (4790383, SmarteX™) were installed. The water tank, piping, connectors, and valves were disinfected with 80 % ethanol before use (EA043, ChemSupply). IBRs and coupons were cleaned by dry heat sterilization using thermal treatment at 121 °C for 15 min.

### 2.2 Experimental setup

The experimental sampling was divided into two stages: (1) biofilm establishment and (2) operational. The entire experiment was conducted twice with the model plumbing system disinfected and then reseeded with potable water for the second experiment, as described. Dates of the experiment were March 2021 to August 2021 and December 2021 to May 2022.

#### 2.2.1 Colonization phase

60 L of potable water that had been previously found to be positive for culturable *L. pneumophila* sg1, *Acanthamoeba* group I and *V. vermiformis* [26] was collected from a shower located in a commercial building and added to the water tank (Fig. 1). The sample contained no residual chlorine (free chlorine was measured using Mobile WaterLink® Spin Touch®, SpinDisk™ Reagent Cartridge Single Use Treated Water Series DW13 (LaMotte Pacific Pty. Ltd.)) and no additional disinfectant was added at any stage during the study. An additional 100 L of the same potable water sample was vacuum filtered [onto 47 mm diameter 0.2 μm polycarbonate membrane (GTTP04700, Isopore™)] and the harvested residue was added to the initial 60L. The final concentration of the targeted microbes was confirmed through microbial analysis (*L. pneumophila* sg1: ≈ 10^4^ GU/mL, *Acanthamoeba*: ≈ 10^3^ GU/mL and *V. vermiformis*: ≈ 10^3^ GU/mL). The system was then left stagnant, without further addition of any nutrients or microbial inoculum, for a period of ≈ five months (147 days) at 37 ± 2 °C to allow the biofilm to establish. This time period was chosen based on previous literature to ensure the establishment of a biofilm community that included *Legionella* [27] this was confirmed by testing the biofilm formed prior to commencing the operational phase (described below).

#### 2.2.2 Operational phase

After 147 days of water stagnation the model entered the operational phase. During this period, the effect of unidirectional hydraulic flow was tested fortnightly for 28 days. One IBR was used to represent low usage and was flushed once-a-week. The other reactor was used to represent high usage and was flushed once-a-day. In each flushing cycle, ≈70 L water (flow rate: ≈3.5 L/min) was drained out from each IBR, which is roughly equal to the average amount of water utilised per shower [28].

### 2.3 Sample collection and processing

After the biofilm colonization phase (day 147), and at day 14 and day 28 of the operational phase, four coupons were collected from each IBR. Each coupon was placed in a sterile 50 mL tube with 5 mL of 1X phosphate-buffered saline (PBS; P4417, Sigma-Aldrich) then scraped using sterile polyurethane-tipped swabs (CleanFoam® TX751B, Texwipe®) followed by 15 min vigorous shaking (wrist action shaker: 896331, Griffin & George Ltd.) and 5 min of sonication in an ultrasonic water bath (895, Cooper Surgical, Inc.) to detach the biofilms. This destructive sampling approach for the biofilm limited the maximum number of samples. The sampling time points were chosen as they were considered most likely to show changes in *Legionella* concentrations based on previous literature [27]. Water sampling was performed after completion of the colonization phase and at day 7, 14, 21 and 28 of the operational phase. A 100 mL water sample was collected from each IBR in triplicate prior to flushing. The water sample was vacuum filtered through a 47 mm diameter, 0.2 μm pore size, polycarbonate membrane (GTTP04700, Isopore™). The filter membrane was resuspended in 5 mL of 1X PBS and vortexed for 10 min to dislodge microbes. Both biofilm and water suspensions were further analysed to characterize surface adherent and planktonic microbes, respectively.

### 2.4 Molecular testing

Quantification of *Legionella*, *L. pneumophila*, *Acanthamoeba*, and *V. vermiformis* was conducted using quantitative qPCR. Standard qPCR assays were used to detect and quantify 16 rDNA and *mip* genes of *Legionella* and L. *pneumophila*, respectively [24]. To quantify *Acanthamoeba* and *V. vermiformis* the 18S rDNA gene was used for amplification [29,30]. Briefly, 1 mL biofilm/water suspension was processed for DNA extraction using the Aquadien™ kit (3578121, BIO-RAD Laboratories Ltd.). The qPCR reaction mixture contained 1X PCR reaction buffer (2X SsoAdvanced™ universal probes supermix:172–5281, Bio-Rad Laboratories Ltd.), microbe-specific oligos (Bio-Rad Laboratories Ltd.) and template DNA. To detect the presence of environmental inhibitors of PCR, both the purified and the 10 times diluted DNA samples were used as template [14,26]. Each template DNA was amplified in triplicate using Rotor-Gene Q thermal cycler (Qiagen Ltd.). Primers and fluorescence labelled probes used in this study are listed in Table S1. Quantification of template DNA was done using a standard curve comprising a concentration range of 10^2^ to 10^9^ copies per reaction. Synthetic DNA fragments (gBlocks, IDT™) of 16S rDNA *Legionella* (Accession Number CP021281), *mip L. pneumophila* (Accession Number KR902705), 18S rDNA *Acanthamoeba castellanii* (Accession Number U07413) and 18S rDNA *V. vermiformis* (Accession Number KT185625) genes were used as standards and a positive control [26]. Gene markers of biofilm-associated microorganisms and planktonic microorganisms were estimated in genomic units per mL (GU/mL) and genomic units per cm^2^ (GU/cm^2^), respectively. It is estimated that the limit of detection for both *Legionella* and L. *pneumophila* was 35 GU/reaction, whereas it was 40 GU/reaction for *Acanthamoeba* and 44 GU/reaction for *V. vermiformis*.

### 2.5 Quantification of total viable *Legionella* population

Total viable *Legionella* and L. *pneumophila* (which include both alive potentially culturable and VBNC cells) were quantified using viability based flow cytometry-cell sorting and qPCR (VFC + qPCR) [25]. Briefly, 300 μL sample was mixed with 200 μL filter sterilized staining buffer (1 mM EDTA and 0.01 % tween-20 in 1X PBS, pH 7.4 ± 0.1). Using the Becton Dickinson (BD™) cell viability kit (349480, BD™), 420 nM of thiazole orange (TO: λ_(excitation)_/λ_(emission)_: 512/533 nm) and 48 μM propidium iodide (PI; λ_(excitation)_/λ_(emission)_: 537/618 nm) was added and incubated at 5 °C for 15 min. Then, cells were analysed on a BD™ FACSAria™ Fusion instrument and segregated into alive (potentially culturable: TO-stained fraction), dead (PI-stained fraction), and injured (potentially VBNC: TO-stained fraction) cell populations. Both alive and injured cell fractions were isolated for further analysis. These fractions were subjected to DNA extraction and quantification of *Legionella* and L. *pneumophila* gene markers as described above.

### 2.6 Microbiological analysis

Standard culture methods were used to detect and quantify biofilm-associated microorganisms (surface adherent) and planktonic (floating in water) culturable bacteria and amoebae. Culturable *Legionella* and L. *pneumophila* were grown and quantified according to standard guidelines [23,31]. Briefly, heat treated (50 ± 1 °C for 30 ± 2 min), acid treated (HCl–KCl buffer treatment for 5 ± 0.5 min) and untreated samples were plated on *Legionella* agar (CM1203, Oxoid Ltd.) supplemented with GVPC (glycine, vancomycin, polymyxin B and cycloheximide: SR0152, Oxoid Ltd.) and *Legionella* growth supplement (α-ketoglutarate, buffer/potassium hydroxide, ferric pyrophosphate, and _l_-cysteine: SR0110C, Oxoid Ltd.). *Legionella-*like colonies were enumerated after 3–7 days of incubation at 37 °C. These presumed *Legionella* were confirmed, and the species serologically identified using *Legionella* latex agglutination test kit (DR0800, Oxoid Ltd.). Heterotrophic plate counts (HPC) were obtained by culture on R_2_A agar (CM0906, Oxoid Ltd.) after 2, 5 and 7 days of incubation at 35 °C. Culturable Gram-negative bacteria and *Pseudomonas* were enumerated after growth on MacConkey (CM0007, Oxoid Ltd.) and cetrimide agar (CM059, Oxoid Ltd.), respectively. Colonies were counted after 2–5 days of incubation at 37 °C. Detection of culturable amoebae was achieved by growing the samples on heat-inactivated (57 °C for 45 min) *Escherichia coli* American Type Culture Collection 700891™ supplemented 1.5 % non-nutrient agar (Eco-NNA: CM0003, Oxoid Ltd.) at 25 °C for 14 days. The growth of amoebae was examined daily with the aid of an inverted light microscope (AMEFC4300, EVOS™ FL, ThermoFisher Scientific).

### 2.7 Microscopic analysis

Fluorescence *in situ* hybridization (FISH) with oligonucleotide probes combined with confocal laser scanning microscopy was used to examine the microbial composition of the biofilms. In the FISH assay, Alexa Fluor 488 labelled LEG705 [32], Alexa Fluor 546 labelled EUB338 [33] and Alexa Fluor 647 labelled EUK1209 [34] fluorogenic oligonucleotide probes (Invitrogen™, Table S1) were used for the detection of *Legionella*, eubacteria, and eukaryotic microbes, respectively. In this assay, paraformaldehyde fixed biofilm samples were dehydrated in an ethanol series (50 %, 80 % and 90 %), then covered with hybridization buffer (0.9 mM NaCl, 0.01 % SDS and 0.02 M Tris-HCl, pH 7.6) containing 100 ng of each fluorogenic oligonucleotide probe and incubated at 55 °C under humid conditions for 100 min. After final washing and drying steps, samples were mounted with CitiFluor™ AF1 (17970–25, Electron Microscopy Sciences) and images were acquired by confocal microscopy (LSM 880 fast airyscan confocal, Zeiss) using oil immersion objective (C Plan-Apochromat 63x/1.4 oil DIC M27, Zeiss) [35]. Moreover, the same imaging settings were used when comparing the degree of labelling between samples. The processing of captured images was conducted using Fiji software (https://imagej.net/software/fiji/).

### 2.8 Data analysis

The log transformed data are depicted as mean ± standard deviation of six to eighteen independent replicates. Statistical analyses and graphical representation were performed using R language computer program agricolae (version 1.3–5) and ggplot2 (version 3.3.6) packages in R environment [36,37]. To compare daily and weekly flushing bioreactors (i.e., biofilm coupons at day 0, 14 and 28 and water at day 0, 7, 14, 21 and 28), data was log transformed and normality was checked by quantile-quantile (q-q) plots and Shapiro-Wilk test. Then, an ANOVA was performed followed by a Tukey's honestly significant difference (HSD) and least significant difference (LSD) tests. Correlation between flushing and other microbiological parameters were computed by comparing the frequency of flushing events (both daily and weekly events are combined) versus microbiological factors (Legionella or another microbe). Here flushing events of both daily and weekly events are combined as number of flushing events 1, 2, 3, 4, 7, 14, 21, 28 for water and 2, 4, 7, 14, 28 for coupons. Similarly, when microbiology factors of both daily and weekly events was combined the data was non-normal, this was confirmed with quantile-quantile (q-q) plots and Shapiro-Wilk tests.

## 3 Results

### 3.1 Biofilm colonization

After 147 days in the colonization phase, visual inspection found that the biofilm depositions were relatively homogenous between and along the coupons. In the stagnant water suspended semi-solid residues were observed. The microbial population developed on coupons contained a diverse mixture of heterotrophic aerobic bacteria, Gram-negative bacteria, and amoebae. Each coupon was colonized by high numbers of *Legionella* (≈6.7 log_10_) and culturable heterotrophic bacteria (≈4.7 log_10_) (Table 1). The difference between total *Legionella* (VBNC, culturable and dead) estimated by qPCR and culturable *Legionella* counted by classical culturing method was ≈2 log_10_. Culturable *Pseudomonas* was absent from both biofilm and water phases. Unlike *Legionella* and culturable heterotrophic bacteria, the number of planktonic amoebae in water samples were significantly higher than biofilm-associated amoebae (Table 1, Table 2).

**Table 1:** Influence of flushing events on biofilm-associated bacteria and amoebae.

Samples	*Legionella* log (GU/cm^2^)			Culturable Bacteria log (CFU/cm^2^)		Amoebae log (GU/cm^2^)	
	Total *Legionella*	Alive (and culturable) *Legionella*	VBNC *Legionella*	*Legionella*	Heterotrophic plate count	*Acanthamoeba*	*Vermamoeba vermiformis*
Colonization phase (Day 0)	6.80 ± 0.18 (a)	5.22 ± 0.19 (a)	3.85 ± 0.08 (b)	4.73 ± 0.04 (a)	4.78 ± 0.05 (a)	3.36 ± 0.20 (e)	3.60 ± 0.13 (c)
Once-a-week flushing (Day 14)	5.70 ± 0.22 (b)	4.63 ± 0.13 (b)	2.88 ± 0.08 (d)	4.05 ± 0.03 (b)	4.29 ± 0.05 (b)	5.40 ± 0.16 (a)	3.41 ± 0.12 (d)
Once-a-week flushing (Day 28)	5.55 ± 0.15 (b)	4.36 ± 0.11 (c)	3.02 ± 0.12 (d)	3.738 ± 0.06 (c)	4.22 ± 0.03 (b)	5.16 ± 0.12 (b)	3.67 ± 0.13 (c)
Once-a-day flushing (Day 14)	5.60 ± 0.17 (b)	3.82 ± 0.11 (d)	3.99 ± 0.06 (a)	3.65 ± 0.04 (c)	4.11 ± 0.05 (c)	4.81 ± 0.07 (c)	6.27 ± 0.16 (a)
Once-a-day flushing (Day 28)	4.47 ± 0.18 (c)	2.79 ± 0.12 (e)	3.311 ± 0.18 (c)	2.20 ± 0.12 (d)	3.80 ± 0.06 (d)	4.20 ± 0.09 (d)	5.81 ± 0.10 (b)

The log transformed data are represented as mean ± standard deviation.

The same alphabetic letter in a column represents statistical similarities at *p* < 0.001 according to Tukey's HSD test.

**Table 2:** Influence of flushing events on planktonic bacteria and amoebae.

Samples	*Legionella* log (GU/mL)			Culturable Bacteria log (CFU/mL)		Amoebae log (GU/mL)	
	Total *Legionella*	Alive (and culturable) *Legionella*	VBNC *Legionella*	*Legionella*	Heterotrophic plate count	*Acanthamoeba*	*Vermamoeba vermiformis*
Colonization phase (Day 0)	5.89 ± 0.19 (a)	3.37 ± 0.16 (a)	3.02 ± 0.15 (a)	3.79 ± 0.10 (a)	4.42 ± 0.13 (a)	3.93 ± 0.11 (a)	4.83 ± 0.13 (a)
Once-a-week flushing (Day 07)	3.83 ± 0.06 (b)	2.41 ± 0.10 (b)	1.67 ± 0.19 (c)	2.52 ± 0.10 (b)	3.37 ± 0.19 (b)	2.27 ± 0.10 (b)	2.51 ± 0.08 (d)
Once-a-week flushing (Day 14)	3.47 ± 0.17 (c)	2.30 ± 0.11 (b)	1.80 ± 0.11 (c)	1.93 ± 0.07 (c)	2.83 ± 0.14 (d)	2.07 ± 0.04 (c)	2.38 ± 0.14 (d)
Once-a-week flushing (Day 21)	2.66 ± 0.06 (d)	1.47 ± 0.10 (d)	1.00 ± 0.10 (e)	1.38 ± 0.08 (d)	2.71 ± 0.07 (d)	1.84 ± 0.10 (d)	2.77 ± 0.13 (c)
Once-a-week flushing (Day 28)	2.58 ± 0.26 (de)	1.39 ± 0.10 (d)	0.90 ± 0.08 (e)	1.39 ± 0.14 (d)	2.67 ± 0.14 (d)	1.48 ± 0.13 (e)	2.98 ± 0.10 (b)
Once-a-day flushing (Day 07)	2.47 ± 0.09 (de)	1.86 ± 0.12 (c)	2.17 ± 0.12 (b)	1.55 ± 0.13 (d)	3.05 ± 0.06 (c)	2.17 ± 0.16 (bc)	2.48 ± 0.12 (d)
Once-a-day flushing (Day 14)	2.40 ± 0.13 (e)	1.86 ± 0.12 (c)	2.16 ± 0.12 (b)	1.46 ± 0.12 (d)	2.79 ± 0.09 (d)	1.85 ± 0.07 (d)	2.36 ± 0.12 (d)
Once-a-day flushing (Day 21)	2.15 ± 0.06 (f)	1.43 ± 0.05 (d)	1.73 ± 0.05 (c)	1.17 ± 0.11 (e)	2.00 ± 0.06 (e)	1.55 ± 0.12 (e)	2.47 ± 0.16 (d)
Once-a-day flushing (Day 28)	1.50 ± 0.07 (g)	0.88 ± 0.08 (e)	1.18 ± 0.08 (d)	0.89 ± 0.08 (f)	2.04 ± 0.09 (e)	0.83 ± 0.09 (f)	1.87 ± 0.11 (e)

The log transformed data are represented as mean ± standard deviation.

The same alphabetic letter in a column represents statistical similarities at *p* < 0.001 according to Tukey's HSD test.

### 3.2 *Legionella* and flow dynamics

Statistical analysis clearly showed that all four categories of the *Legionella* population screened in this study were significantly affected by flushing frequency (Table 1, Table 2 and Fig. 3, Fig. 4). Interesting, the acid and heat pre-treatment options used in the standard culture method affected the recovery of viable *Legionella.* Either pre-treatment method reduced the culturable *Legionella* to undetectable levels in all cases for day 7, 14, 21 and 28 days, both in the water and biofilm. Therefore, pre-treatment steps recommended by standard culturing methods were omitted and bacterial colonies were characterized by serology and molecular identification assays described in 2.6.

#### 3.2.1 Biofilm-associated *Legionella*

**Fig. 2:**
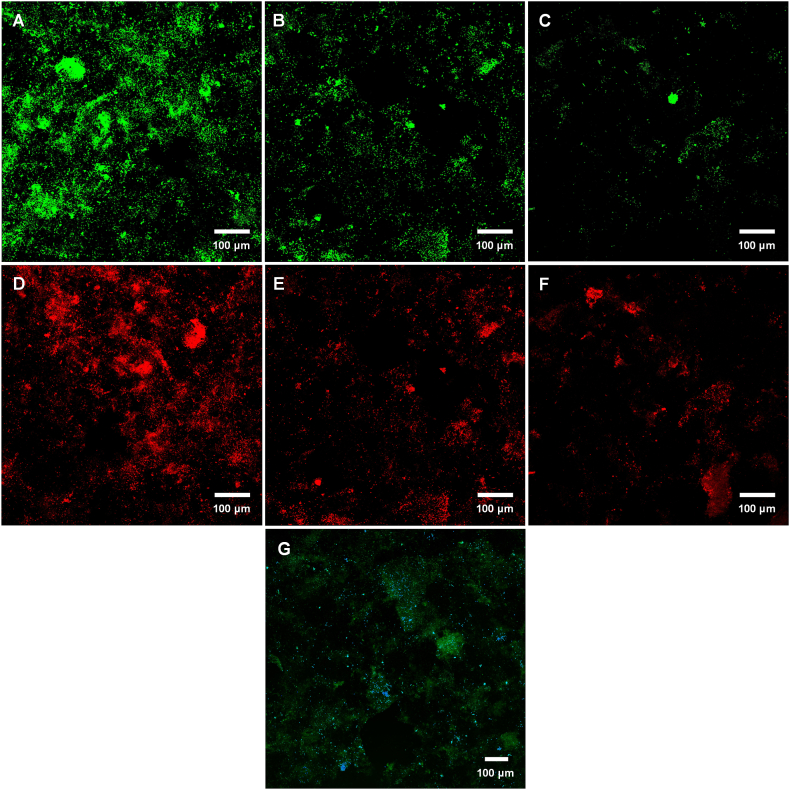
Fluorescence *in situ* hybridisation images of biofilms from the model plumbing system stained with *Legionella* (green coloured), eubacteria (red coloured), and eukaryotic (blue coloured) specific probes. Figures A, D and G show that the establishment phase resulted in the formation of well-established biofilm consisting of *Legionella* (green coloured), eubacteria (red coloured) and eukaryotic microorganisms (blue coloured). In comparison to once-a-week flushing for 28 days (B and E), once-a-day flushing for 28 days (C and F) decreased the population of biofilm-associated *Legionella* and eubacteria. The bar represents 100 μm.

**Fig. 3:**
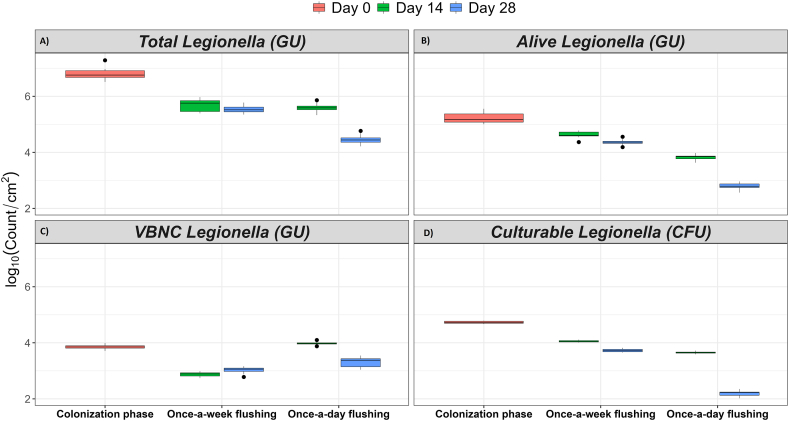
The effect of flushing events on the biofilm-associated **(A)** total, **(B)** alive (potentially culturable), **(C)** VBNC and **(D)** culturable *Legionella*. The log transformed data are shown as mean ± standard deviation of nine to eighteen replicates. GU: genomic unit quantified by qPCR assay; CFU: colony forming unit estimated by standard culturing method. Pink colour: colonization phase, green colour: day 14, and blue colour: day 28. The bar across each box represents standard deviation.

**Fig. 4:**
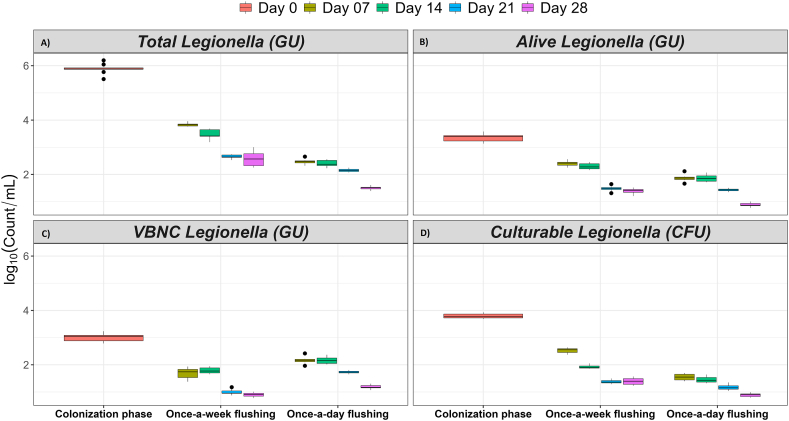
The effect of flushing events on the planktonic **(A)** total, **(B)** alive (potentially culturable), **(C)** VBNC and **(D)** culturable *Legionella*. The log transformed data is shown as mean ± standard deviation of nine replicates. GU: genomic unit quantified by qPCR assay; CFU: colony forming unit estimated by standard culturing method. Pink colour: colonization phase, olive colour: day 7, green colour: day 14, blue colour: day 21, and dark pink colour: day 28. The bar across each box represents standard deviation.

The effect of high and low frequency flushing events on biofilm-associated *Legionella* is shown in Fig. 3, Fig. 4. The incidence of flushing significantly influenced the populations of total (Fig. 3, Fig. 4A), alive (Fig. 3, Fig. 4B), VBNC (Fig. 3, Fig. 4C) and culturable (Fig. 3, Fig. 4D) *Legionella*. Total (both viable and dead), alive (potentially culturable) and culturable *Legionella* populations were significantly reduced with increased flushing events. In contrast to weekly flushing, daily flushing significantly (*p* < 0.001) reduced total *Legionella* by 19.4 % on day 28 (Fig. S1). Likewise, daily flushing significantly decreased the population of alive *Legionella* by 17.5 % and 36 % on day 14 and 28, respectively, as compared to weekly flushing (Table 1 and Fig. S2). The population of culturable *Legionella* was also sensitive to flushing events. The daily flushing events resulted in a reduction of culturable *Legionella* by 9.9 % on day 14 and 41 % on day 28 (Table 1 and Fig. S4). Similarly, flushing frequency was negatively correlated with total *Legionella* (ρ = −0.651, *p* < 0.001), alive *Legionella* (ρ = −0.955, *p* < 0.001) and culturable *Legionella* (ρ = −0.939, *p* < 0.001). This showed that once-a-day flushing produced a greater reduction compared with once-a-week flushing. This analysis was supported by the FISH micrographs which at the commencement of the operational phase showed (Fig. 2A–D and G) the formation of well-established biofilm consisting of *Legionella* (green coloured), eubacteria (red coloured) and eukaryotic microorganisms (blue coloured). In Fig. 2A, the presence of some areas of increased *Legionella* density demonstrated that *Legionella* developed compact and well-structured biofilms. In comparison, biofilms formed under weekly (Fig. 2B and E) and daily (Fig. 2C and F) flushing (for 28 days), clearly illustrate that daily flushing decreased the amount of biofilm-associated *Legionella*. On the other hand, daily flushing significantly increased VBNC *Legionella* by 27.6 % on day 14 and 8.8 % on day 28 (Table 1 and Fig. S3). Likewise, flushing was positively correlated to VBNC *Legionella* (ρ = 0.696, *p* < 0.001). Overall, Spearman's analysis demonstrated that high flushing frequency decreased the amount of alive and culturable *Legionella* but increased the quantity of VBNC *Legionella*.

#### 3.2.2 Planktonic *Legionella*

Fig. 4 shows boxplots of flushing events and the amount of planktonic *Legionella* present in the water samples. The plots show that the populations of total, alive and culturable *Legionella* were higher in the IBR that was flushed weekly compared with the IBR flushed daily. Flushing daily significantly reduced the total amount of *Legionella* by 35.4 %, 30.8 %, 19 % and 41 % on day 7, 14, 21 and 28, respectively, compared with the samples collected on the same days from the IBR that was flushed weekly (Table 2 and Fig. S5). In comparison with weekly flushing, daily flushing significantly reduced the population of alive *Legionella* by 19.2 % on day 14 and 36.5 % on day 28 (Table 2 and Fig. S6). The daily flushing events resulted in a reduction of culturable *Legionella* by 38.6 %, 24.4 %, 14.9 %, and 36.2 % on day 7, 14, 21, and 28, as compared with weekly flushing (Table 2 and Fig. S8). Likewise, flushing frequency negatively correlated with total *Legionella* (ρ = −0.947, *p* < 0.001), alive *Legionella* (ρ = −0.706, *p* < 0.001) and culturable *Legionella* (ρ = −0.816, *p* < 0.001). It clearly showed that high flushing frequency decreased the amount of alive and culturable *Legionella*. However, daily flushing, when compared with weekly flushing, significantly increased the population of VBNC *Legionella* by 22.8 % on day 7, 16.8 % on day 14, 42.4 % on day 21, and 24 % on day 28 (Table 2 and Fig. S7). Similarly, flushing positively correlated to planktonic VBNC *Legionella* (ρ = 0.802, *p* < 0.001). The combination of detection methods demonstrated that overall, the once-a-day flushing resulted in a statistically significant reduction in the alive *Legionella*, but an increase in VBNC *Legionella* compared with the once-a-week flushing.

### 3.3 Culturable heterotrophic bacterial population and flow dynamics

**Fig. 5:**
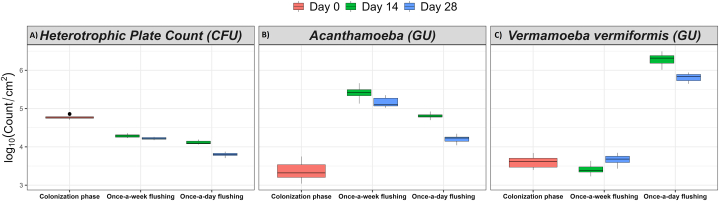
The effect of flushing events on the biofilm-associated **(A)** heterotrophic bacteria and amoebae, **(B)***Acanthamoeba* and **(C)** V*ermamoeba vermiformis*. The log transformed data is shown as mean ± standard deviation of six to twelve replicates. GU: genomic unit quantified by qPCR assay; CFU: colony forming unit estimated by standard culturing method. Pink colour: colonization phase, green colour: day 14, and blue colour: day 28. The bar across each box represents standard deviation.

**Fig. 6:**
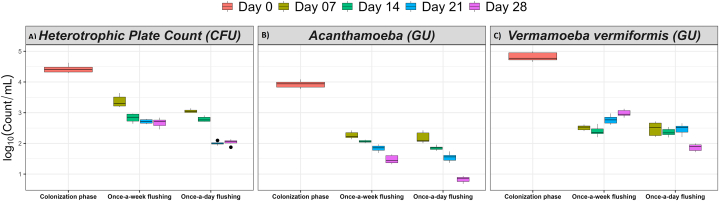
The effect of flushing events on the **(A)** planktonic heterotrophic bacteria and amoebae, **(B)***Acanthamoeba* and **(C)** V*ermamoeba vermiformis*. The log transformed data is shown as mean ± standard deviation of six replicates. GU: genomic unit quantified by qPCR assay; CFU: colony forming unit estimated by standard culturing method. Pink colour: colonization phase, olive colour: day 7, green colour: day 14, blue colour: day 21, and dark pink colour: day 28. The bar across each box represents standard deviation.

The effect of flushing frequency on biofilm-associated and planktonic heterotrophic culturable bacteria is represented in Fig. 5, Fig. 6A, respectively. It was found that over time the once-a-day flushing resulted in a consistent decline in the HPC. In contrast to weekly flushing, daily flushing significantly (*p* < 0.001) reduced biofilm-associated heterotrophic bacteria by 9.9 % on day 28 (Table 1 and Fig. S9). The daily flushing events resulted in a reduction of planktonic heterotrophic bacteria by 9.2 % on day 7 and 23.8 % on day 28, as compared with weekly flushing (Table 2 and Fig. S10). Similarly, Spearman's analysis demonstrated that flushing frequency negatively correlated with both the biofilm-associated (ρ = −0.942, *p* < 0.001) and planktonic (ρ = −0.683, *p* < 0.001) heterotrophic bacteria.

These results revealed that high frequency flushing significantly reduced the heterotrophic bacterial counts. The morphological characteristics of the bacterial colonies were analysed to assess the diversity and community structure of planktonic and biofilm-associated culturable heterotrophic bacteria. The biofilm-associated heterotrophic bacterial population showed a stable and consistent community over time and was apparently not affected by the flushing events, as the diversity remained the same and low throughout the operational phase. However, strong bacterial community shifts within planktonic heterotrophic bacteria were seen in daily flushing IBR. Similarly, the pattern of planktonic Gram-negative bacteria showed striking shifts after daily flushing. As observed in the colonization phase, culturable *Pseudomonas* was absent from both water and biofilm in all phases.

### 3.4 Amoebae and flow dynamics

Both culturing and the qPCR assays successfully detected and quantified planktonic and biofilm-associated *Acanthamoeba* and *V. vermiformis*. These results clearly showed that the amoebae population was significantly affected by the flushing frequency.

#### 3.4.1 Acanthamoeba

The effect of flushing frequency on biofilm-associated and planktonic *Acanthamoeba* is depicted in Fig. 5, Fig. 6B, respectively. Daily flushing significantly reduced biofilm-associated *Acanthamoeba* by 10.8 % on day 14 and 18.5 % on day 28 (Table 1 and Fig. S11); and planktonic *Acanthamoeba* by 10.6 %, 15.7 % and 43.7 % on day 14, 21 and 28 (Table 2 and Fig. S12), respectively, compared with the IBR that was flushed once-a-week. Likewise, flushing frequency was negatively related to both the biofilm-associated (ρ = −0.949, *p* < 0.001) and planktonic (ρ = −0.717, *p* < 0.001) *Acanthamoeba*. It demonstrated that once-a-day flushing decreased the population of both planktonic and biofilm-associated *Acanthamoeba*.

#### 3.4.2 Vermamoeba vermiformis

Fig. 5, Fig. 6C show the effect of flushing frequency on biofilm-associated and planktonic *V. vermiformis*, respectively. Under daily flushing conditions, the population of the biofilm-associated *V. vermiformis* increased by 83.9 % on day 14 and 58.3 % on day 28, compared with their respective time samples from the once-a-week flushing IBR (Table 1 and Fig. S13). In the case of planktonic *V. vermiformis*, the amoebae population initially remained unaltered for 14 days; however, the population dropped by 10.8 % on day 21 and 37.1 % on day 28 in the IBR that was flushed daily compared with the corresponding time samples from the IBR that was flushed weekly (Table 2 and Fig. S14). Flushing frequency was positively correlated to biofilm-associated *V. vermiformis* (ρ = 0.706, *p* < 0.001), but was negatively correlated to planktonic *V. vermiformis* (ρ = −0.362, *p* < 0.001). This highlights that once-a-day flushing increased the quantity of biofilm-associated *V. vermiformis* but decreased planktonic *V. vermiformis*.

### 3.5 Relationship between *Legionella* and microbial flora

Culturable *Legionella* and heterotrophic plate count were positively correlated (biofilm-associated: ρ = 0.929, *p* < 0.001 and planktonic: ρ = 0.802, *p* < 0.001). Similarly, alive *Legionella* were significantly correlated with *Acanthamoeba* (biofilm-associated: ρ = 0.917, *p* < 0.001 and planktonic: ρ = 0.894, *p* < 0.001). In addition, biofilm-associated VBNC *Legionella* were positively correlated with biofilm-associated *V. vermiformis* (ρ = 0.848, *p* < 0.001), whereas planktonic VBNC *Legionella* were positively correlated with planktonic *Acanthamoeba* (ρ = 0.526, *p* < 0.001). It demonstrated that VBNC *Legionella* and amoebae populations increased concurrently. It can be hypothesized that the daily flushing had an impact on culturable *Legionella*, which were transformed into VBNC *Legionella* via intracellular replication within host amoebae. Furthermore, the FISH micrographs (Fig. 2G) demonstrated that *Legionella* appeared in distinct clusters and often associated with protozoa, suggesting a strong association between *Legionella* and protozoa in biofilms.

## 4 Discussion

Various chemical and physical water disinfection protocols are designed to control *Legionella* in engineered water systems [38]. However, in the real world none of these disinfection procedures can achieve total eradication of *Legionella* [[39], [40], [41]]. From the water source to the point of utilization, the concentration of chemical disinfectants fluctuates with disinfection decay accelerated by biofilms and water stagnation [42]. Thermal disinfection is another physical approach used to control *Legionella* in building water systems [43]. However, the presence of dead ends and dead legs (which are not reached by hot water used for disinfection treatment), biofilms (which provide protection) and the development/selection of thermotolerant strains results in frequent failure of thermal disinfection [15,44]. Extended water stagnation and water aging in buildings are significant factors influencing *Legionella* proliferation [15]. This is especially topical due to COVID-19 lockdowns, as well as the increased interest in green buildings. Green buildings use available strategies of water conservation including plumbing fixtures that reduce usage and flow of potable water [45,46]. These strategies reduce water usage by increasing the hydraulic retention time of the building plumbing system. In summary, these building plumbing systems have higher surface area to volume ratios, water stagnation, variable hydraulic regimes, and water temperature [47]. This increased half-life of water in the building system and permits increased decay of chemical disinfectants as shown by a study conducted in the USA that consistently detected lower concentrations of chlorine in green building plumbing systems [17].

Previous studies have indicated that in building plumbing systems the removal of water stagnation points reduces the risk of legionellosis [19,43]. However, there are some studies that did not find any positive relationship between *Legionella* persistence and water stagnation [21,22]. Some authors suggest that water circulation in piping networks support the colonization of *Legionella* [22]. This positive association of water circulation with *Legionella* contamination is justified by the “nutrient and oxygen supply hypothesis”, which suggests that circulating water evenly distributes nutrients and microbes which accelerates microbial growth in the building plumbing system [22]. In the present study, the model plumbing system was designed with a unidirectional water flow to prevent the recirculation and mixing of residual water. This simulates the water stagnation occurring at plumbing outlets.

During water stagnation in building plumbing systems, chemical, physical and biological parameters of potable water are interlinked and affect each other [48]. In this study, we only focused on biological quality parameters. We examined how flushing events and water stagnation influenced the growth and persistence of *Legionella* and host free-living amoebae in the building plumbing system. To do this both the routine culturing and qPCR assays were complimented with the use of VFC + qPCR to quantify VBNC *Legionella* in both biofilm and water samples. The concentrations of *Legionella* determined via qPCR were between 0.7 and 2.1 log_10_ greater than culturable *Legionella*. This discrepancy can be explained by the population of VBNC *Legionella* estimated by VFC + qPCR and dead *Legionella*. Using the VFC + qPCR assay the alive (potentially culturable) *Legionella* was also estimated, and this approximately was the same concentration of *Legionella* estimated by culture.

It is important to note that water and biofilm samples collected during the operational phase were sensitive to both the acid and heat pre-treatment recommended by ISO11731:2017–05 [23]. These pre-treatment steps have been shown to successfully increase the sensitivity of *Legionella* detection in samples with high levels of other bacteria. However, previous work has shown that sample handling and both thermal and acid treatment steps are responsible for ≈30 % transformation of culturable *Legionella* to VBNC *Legionella* [25,49]. Therefore, given the high concentration of *Legionella* present in this model study, these pre-treatment steps were skipped. It also supports the assertions that previous studies that solely used *Legionella* culture may underestimate the real burden of *Legionella*.

In the real world, building water systems consist of plumbing pipes, fixtures, and devices from point of entry to point of delivery [48]. It is very difficult to simulate such a complex and highly variable system in a laboratory. Our study designed and validated a simplified plumbing system model with a naturally formed biofilm (Fig. 1). This model plumbing system was capable of developing a stable microbial ecosystem in non-supplemented shower water contaminated with *Legionella* and amoebae. The microflora constituting this ecosystem predominantly consisted of *Legionella*, culturable heterotrophic bacteria, *Acanthamoeba* and *V. vermiformis*. The model was shown to be able to harbor very high numbers of *Legionella*. It models natural microbial communities typically present within actual building plumbing systems [14,50]. Our designed plumbing model system is a valuable tool to study colonization and persistence of *Legionella* in engineered water systems. However, given the simplified design of the plumbing model, future research is needed to examine the relationships of these pathogens in real world systems that are significantly more complex due to varying environmental and design parameters and water management approaches (such as the inclusion of disinfectants). Also, another limitation of the current study is the high concentration of *Legionella* used to colonise the model, which may not be representative of a typical potable water distribution system.

Biofilm sloughing is driven by biological and physical factors. To our knowledge, the current study is the first time that (1) biofilm sloughing by water flushing (a physical factor); (2) the effect of water flushing on biofilm-associated microbes and (3) planktonic microflora, have been comprehensively examined using an integrated approach. During the colonization phase (147 days water stagnation), the microbes slough off from loosely attached biofilm and disperse into surrounding stagnant water. The planktonic bacteria and amoebae growing in the stagnant water primarily detached from biofilm (developed on polypropylene disc coupons) by some active biological processes. Generally, biological factors responsible for this detachment are microbial communication mediated dispersal, seeding dispersal, cell division mediated dispersal and nutrient fluctuations mediated dispersal [51].

The results of the operational phase presented in Table 1, Table 2 showed that the lowest concentration of alive (potentially culturable) and culturable *Legionella* was always recovered from the biofilms growing in the high-use IBR. In the case of potable water, it is recognised that flushing events are the most important physical factors responsible for biofilm sloughing [52,53]. It was observed that initially there was little difference in the concentration of total *Legionella* in both high-use and low-use IBRs after 14 days of the operation phase (Table 1). However, culturing results demonstrated that the *Legionella* population decreased by 1.9 log_10_ in the high-use IBR (Fig. 4). By the end of operational phase, biofilm-associated culturable *Legionella* were 1.5 log_10_ greater in the low-use IBR relative to the high-use IBR, suggesting that this difference was induced by flushing events (Table 1 and Fig. 4) and perhaps water stagnation stimulated denser and stronger attachment of biofilm to surfaces. A previous pilot scale study of a plumbing system also suggested that the concentration of *Legionella* increases with water stagnation [16]. This supported the findings from this study that showed once-a-week flushing increased water stagnation time, which resulted in proliferation of both planktonic and biofilm-associated alive and culturable *Legionella*. Alternatively, once-a-day water flushing caused looser attachment (dissociation) of biofilm to surfaces that was then readily removed by flushing. The doubling time of bacteria in potable water is reported to be several days, which supports our argument that during this period of water stagnation bacteria regrow and maintain their population [54,55]. Thus, with a high flushing frequency the water efficiently dislodged biofilm and significantly decreased the number of alive and culturable *Legionella*, however the number of VBNC *Legionella* increased (Table 1 and Fig. 1, Fig. 3). Consequently, the growth of VBNC *Legionella* might have been stimulated by flushing events in a more direct way. After daily flushing, a high number of VBNC *Legionella* were recovered from biofilm matrix in the presence of *V. vermiformis*. Similarly, after daily flushing high concentrations of planktonic VBNC *Legionella* were detected in the water which was highly contaminated with *Acanthamoeba*. *In vitro* studies have shown that VBNC *Legionella* proliferates intracellularly in amoebae [56]. It is also reported that in natural environments, both physiochemical stresses and host protozoans transform culturable *Legionella* into VBNC *Legionella* [15,57]. So, this increase in VBNC *Legionella* could be explained by the fact that daily flushing impacted and promoted the differentiation of culturable *Legionella* into VBNC *Legionella* by the intracytoplasmic division within host amoebae. It is worth noting that biofilm formation is a ‘stress response’ to hostile environments e.g., low nutrients and disinfectant treatments. Flushing introduces both these stressors in building water systems and so may induce the VBNC state. Our findings also suggest that increasing water stagnation flushing did not completely remove *Legionella* and may have stimulated the synthesis of VBNC *Legionella*. This supports the use of routine flushing as a part of a multi-barrier approach to controlling *Legionella* in building water systems.

This study found that culturable heterotrophic bacterial populations negatively correlated with increased incidence of flushing events and positively correlated with increased water stagnation. It showed that water stagnation quickly altered the microbial water quality and substantially increased the number of bacteria. Once-a-day flushing also impacted the structure and diversity of the culturable heterotrophic bacteria population. The municipal water source used for flushing could explain the rapid change in diversity of culturable heterotrophic bacteria. Generally, it is difficult to interpret the public health significance of HPC results because the correlation with OPPPs is debated [58]. Secondly, to our knowledge HPC levels have been not associated with any known disease outbreak and public health concern. However, this study found that HPC levels positively correlated to total, alive and culturable *Legionella* concentrations and water stagnation. These results support a previous survey of residential buildings that also demonstrated a positive correlation between heterotrophic bacterial population and *Legionella* concentration [59].

Free-living amoebae are an important part of plumbing systems [60]. *Acanthamoeba* and *V. vermiformis* are the most common and abundant hosts of *Legionella* [8]. Our results illustrated that the population of amoebae was also affected by water stagnation and flushing. Interestingly, increased incidence of flushing was observed to increase the population of biofilm-associated *V. vermiformis* (Table 1 and Fig. 5C) but decrease the concentration of *Acanthamoeba* (Table 1 and Fig. 5B). This increase in *V. vermiformis* concentration was associated with an increase in VBNC *Legionella*. This is potentially due to the increased transformation of alive *Legionella* into VBNC via *Legionella* intracellular replication within amoeba hosts [9,61]. Secondly, it may have benefited from the delivery of carbon and nutrients to the biofilm after daily flushing. There is another possibility: that once-a-day flushing induced stress on biofilm-associated *Legionella* and other bacteria, which chemotactically attracted amoebae for their protection and genesis of VBNC bacterial cells [61,62]. To our knowledge, effect of water stagnation and water flushing on amoebae growth and proliferation has not yet been investigated. Further studies are required to properly understand how free-living amoebae behave in building plumbing systems under different hydraulic regimes.

By the time potable water reaches the point of delivery within a building, it can be a few hours to several days old. Extended periods of water stagnation are linked to failures of disinfection procedures and increased microbial populations. This study used a model plumbing system to demonstrate that daily water flushing had a significant effect on *Legionella* prevalence in a building plumbing system compared with once-a-week flushing and an extended period of water stagnation. An increased incidence of flushing was statistically significantly associated with a decrease in *Legionella* concentration*.* However, it also demonstrated that once *Legionella* had developed and become incorporated into the biofilm matrix it persisted, and regular flushing was unable to eradicate it. As such, multiple strategies are needed for the management and control of building water systems. This should include the prevention of water stagnation, in combination with additional physical or chemical disinfection approaches.

## Data availability statement

All data generated or analysed during this study are included in this published article and its supplementary information files.

## Ethics statement

This work does not contain any studies conducted with human participants or animals.

## CRediT authorship contribution statement

**Muhammad Atif Nisar:** Writing – original draft, Methodology, Formal analysis, Data curation, Conceptualization. **Kirstin E. Ross:** Writing – review & editing, Supervision, Project administration. **Melissa H. Brown:** Writing – review & editing, Supervision, Conceptualization. **Richard Bentham:** Writing – review & editing. **Giles Best:** Writing – review & editing, Methodology, Conceptualization. **Nicholas S. Eyre:** Writing – review & editing, Methodology. **Sophie C. Leterme:** Writing – review & editing. **Harriet Whiley:** Writing – review & editing, Supervision, Project administration, Methodology, Funding acquisition, Conceptualization.

## Declaration of competing interest

The authors declare that they have no known competing financial interests or personal relationships that could have appeared to influence the work reported in this paper.
