# Supplementary material for: Increased flushing frequency of a model plumbing system initially promoted the formation of viable but non culturable cells but ultimately reduced the concentration of culturable and total Legionella DNA
Source: Heliyon. 2024 Jun 4;10(11):e32334. doi: 10.1016/j.heliyon.2024.e32334 (PMC11200333; doi:10.1016/j.heliyon.2024.e32334)
Supplement: Multimedia component 1 [file mmc1.pdf]

Once-a-day flushing of a model plumbing system significantly reduced colonisation of *Legionella*

Muhammad Atif Nisar [a], Kirstin E. Ross [a], Melissa H. Brown [a, b], Richard Bentham [a], Giles Best [c, d], Nicholas S. Eyre [c], Sophie C. Leterme [a, b, e], Harriet Whiley [a, b *]

[a] College of Science and Engineering, Flinders University, Bedford Park, SA 5042, Australia

[b] ARC Training Centre for Biofilm Research and Innovation, Flinders University, Bedford Park, SA 5042, Australia

[c] College of Medicine and Public Health, Flinders University, Bedford Park, SA 5042, Australia

[d] Flow Cytometry Facility, Flinders University, Bedford Park, SA 5042, Australia

[e] Institute for Nanoscience and Technology, Flinders University, Bedford Park, SA 5042, Australia

*Correspondence: Harriet.Whiley@flinders.edu.au; Tel.: +61-8-7221-8580

List of Tables

Table S1: Sequences of oligos and fluorogenic probes used in this study.

List of Figures

Figure S1: Impact of the flushing frequency on biofilm-associated total *Legionella* quantified by the qPCR assay. The log transformed data are presented as mean ± standard deviation of nine to eighteen replicates. The same alphabetic letter represents statistical similarities at $p < 0.001$ according to Tukey's HSD test. C0: colonization phase, CW14: once-a-week flushing-day 14 sampling, CW28: once-a-week flushing-day 28 sampling, CD14: once-a-day flushing- day 14 sampling, and CD28: once-a-day flushing- day 28 sampling.

Figure S2: Impact of the flushing frequency on biofilm-associated alive *Legionella* (potentially culturable) quantified by a flow cytometry-cell sorting and subsequent qPCR assay. The log transformed data is presented as mean ± standard deviation of nine to eighteen replicates. The same alphabetic letter represents statistical similarities at $p < 0.001$ according to Tukey's HSD test. C0: colonization phase, CW14: once-a-week flushing-day 14 sampling, CW28: once-a-week flushing-day 28 sampling, CD14: once-a-day flushing- day 14 sampling, and CD28: once-a-day flushing- day 28 sampling.

Figure S3: Impact of the flushing frequency on biofilm-associated VBNC *Legionella* quantified by "flow cytometry-cell sorting and qPCR assay". The log transformed data is presented as mean ± standard deviation of nine to eighteen replicates. The same alphabetic letter represents statistical similarities at $p < 0.001$ according to Tukey's HSD test. C0: colonization phase, CW14: once-a-week flushing-day 14 sampling, CW28: once-a-week flushing-day 28 sampling, CD14: once-a-day flushing- day 14 sampling, and CD28: once-a-day flushing- day 28 sampling.

Figure S4: Impact of the flushing frequency on biofilm-associated culturable *Legionella* quantified by "standard culturing assay". The log transformed data is presented as mean ± standard deviation of six to twelve replicates. The same alphabetic letter represents statistical similarities at $p < 0.001$ according to Tukey's HSD test. C0: colonization phase, CW14: once-a-week flushing-day 14 sampling, CW28: once-a-week flushing-day 28 sampling, CD14: once-a-day flushing- day 14 sampling, and CD28: once-a-day flushing- day 28 sampling.

Figure S5: Impact of the flushing frequency on planktonic total *Legionella* quantified by "qPCR assay". The log transformed data is presented as mean ± standard deviation of nine replicates. The same alphabetic letter represents statistical similarities at $p < 0.001$ according to Tukey's HSD test. W0: colonization phase, WW07: once-a-week flushing-day 07 sampling, WW14: once-a-week flushing-day 14 sampling, WW21: once-a-week flushing-day 21 sampling, WW28: once-a-week flushing-day 28 sampling, WD07: once-a-day flushing- day 07 sampling, WD14: once-a-day flushing- day 14 sampling, WD21: once-a-day flushing- day 21 sampling, and WD28: once-a-day flushing- day 28 sampling.

Figure S6: Impact of the flushing frequency on planktonic alive *Legionella* (potentially culturable) quantified by "flow cytometry-cell sorting and qPCR assay". The log transformed data is presented as mean ± standard deviation of nine replicates. The same alphabetic letter represents statistical similarities at $p < 0.001$ according to Tukey's HSD test. W0: colonization phase, WW07: once-a-week flushing-day 07 sampling, WW14: once-a-week flushing-day 14 sampling, WW21: once-a-week flushing-day 21 sampling, WW28: once-a-week flushing-day 28 sampling, WD07: once-a-day flushing- day 07 sampling, WD14: once-a-day flushing- day 14 sampling, WD21: once-a-day flushing- day 21 sampling, and WD28: once-a-day flushing- day 28 sampling.

Figure S7: Impact of the flushing frequency on planktonic VBNC *Legionella* quantified by "flow cytometry-cell sorting and qPCR assay". The log transformed data is presented as mean ± standard deviation of nine replicates. The same alphabetic letter represents

statistical similarities at $p < 0.001$ according to Tukey's HSD test. W0: colonization phase, WW07: once-a-week flushing-day 07 sampling, WW14: once-a-week flushing-day 14 sampling, WW21: once-a-week flushing-day 21 sampling, WW28: once-a-week flushing-day 28 sampling, WD07: once-a-day flushing- day 07 sampling, WD14: once-a-day flushing- day 14 sampling, WD21: once-a-day flushing- day 21 sampling, and WD28: once-a-day flushing-day 28 sampling.

Figure S8: Impact of the flushing frequency on planktonic culturable *Legionella* quantified by "standard culturing assay". The log transformed data is presented as mean ± standard deviation of six replicates. The same alphabetic letter represents statistical similarities at $p < 0.001$ according to Tukey's HSD test. W0: colonization phase, WW07: once-a-week flushing-day 07 sampling, WW14: once-a-week flushing-day 14 sampling, WW21: once-a-week flushing-day 21 sampling, WW28: once-a-week flushing-day 28 sampling, WD07: once-a-day flushing- day 07 sampling, WD14: once-a-day flushing- day 14 sampling, WD21: once-a-day flushing- day 21 sampling, and WD28: once-a-day flushing- day 28 sampling.

Figure S9: Impact of the flushing frequency on biofilm-associated culturable heterotrophic bacteria quantified by "culturing assay". The log transformed data is presented as mean ± standard deviation of six to twelve replicates. The same alphabetic letter represents statistical similarities at $p < 0.001$ according to Tukey's HSD test. C0: colonization phase, CW14: once-a-week flushing-day 14 sampling, CW28: once-a-week flushing-day 28 sampling, CD14: once-a-day flushing- day 14 sampling, and CD28: once-a-day flushing- day 28 sampling.

Figure S10: Impact of the flushing frequency on planktonic culturable heterotrophic bacteria by "culturing assay". The log transformed data is presented as mean ± standard deviation of six replicates. The same alphabetic letter represents statistical similarities at $p < 0.001$ according to Tukey's HSD test. W0: colonization phase, WW07: once-a-week flushing-day 07 sampling, WW14: once-a-week flushing-day 14 sampling, WW21: once-a-week flushing-day 21 sampling, WW28: once-a-week flushing-day 28 sampling, WD07: once-a-day flushing- day 07 sampling, WD14: once-a-day flushing- day 14 sampling, WD21: once-a-day flushing- day 21 sampling, and WD28: once-a-day flushing- day 28 sampling.

Figure S11: Impact of the flushing frequency on biofilm-associated *Acanthamoeba* quantified by "qPCR assay". The log transformed data is presented as mean ± standard deviation of nine to eighteen replicates. The same alphabetic letter represents statistical similarities at $p < 0.001$ according to Tukey's HSD test. C0: colonization phase, CW14: once-

a-week flushing-day 14 sampling, CW28: once-a-week flushing-day 28 sampling, CD14: once-a-day flushing- day 14 sampling, and CD28: once-a-day flushing- day 28 sampling.

Figure S12: Impact of the flushing frequency on planktonic *Acanthamoeba* quantified by "qPCR assay". The log transformed data is presented as mean ± standard deviation of nine replicates. The same alphabetic letter represents statistical similarities at $p < 0.001$ according to Tukey's HSD test. W0: colonization phase, WW07: once-a-week flushing-day 07 sampling, WW14: once-a-week flushing-day 14 sampling, WW21: once-a-week flushing-day 21 sampling, WW28: once-a-week flushing-day 28 sampling, WD07: once-a-day flushing- day 07 sampling, WD14: once-a-day flushing- day 14 sampling, WD21: once-a-day flushing- day 21 sampling, and WD28: once-a-day flushing- day 28 sampling.

Figure S13: Impact of the flushing frequency on biofilm-associated *Vermamoeba vermiformis* quantified by "qPCR assay". The log transformed data is presented as mean ± standard deviation of nine to eighteen replicates. The same alphabetic letter represents statistical similarities at $p < 0.001$ according to Tukey's HSD test. C0: colonization phase, CW14: once-a-week flushing-day 14 sampling, CW28: once-a-week flushing-day 28 sampling, CD14: once-a-day flushing- day 14 sampling, and CD28: once-a-day flushing- day 28 sampling.

Figure S14: Impact of the flushing frequency on planktonic *Vermamoeba vermiformis* quantified by "qPCR assay". The log transformed data is presented as mean ± standard deviation of nine replicates. The same alphabetic letter represents statistical similarities at $p < 0.001$ according to Tukey's HSD test. W0: colonization phase, WW07: once-a-week flushing-day 07 sampling, WW14: once-a-week flushing-day 14 sampling, WW21: once-a-week flushing-day 21 sampling, WW28: once-a-week flushing-day 28 sampling, WD07: once-a-day flushing- day 07 sampling, WD14: once-a-day flushing- day 14 sampling, WD21: once-a-day flushing- day 21 sampling, and WD28: once-a-day flushing- day 28 sampling.

Table S1: Sequences of oligos and fluorogenic probes used in this study

Name	Sequence and fluorogenic signal (5′ to 3′)	Assay conditions
***Legionella* 16S rDNA gene specific qPCR primers and probe [1]**		
Forward Primer	GGAGGGTTGATAGGTTAAGAGCT	Stage I: one cycle of 95°C for 3 min<br>Stage II: 43 cycles of 95°C for 20 s and 60°C for 60 s
Reverse Primer	CCAACAGCTAGTTGACATCGTTT	
Probe	6 FAM–AGTGGCGAAGGCGGCTACCT–Iowa Black FQ	
***L. pneumophila mip* gene specific qPCR primers and probe [1]**		
Forward Primer	CCGATGCCACATCATTAGC	Stage I: one cycle of 95°C for 3 min<br>Stage II: 43 cycles of 95°C for 20 s and 60°C for 60 s
Reverse Primer	CCAATTGAGCGCCACTCATAG	
Probe	6 FAM–TGCCTTTAGCCATTGCTTCCG–Iowa Black FQ	
***Acanthamoeba* 18S rDNA gene specific qPCR primers and probe [2]**		
Forward Primer	CCCAGATCGTTTACCGTGAA	Stage I: one cycle of 95°C for 3 min<br>Stage II: 40 cycles of 95°C for 20 s and 63°C for 60 s
Reverse Primer	TAAATATTAATGCCCCCAACTATCC	
Probe	6 FAM–CTGCCACCGAATACATTAGCATGG–Iowa Black FQ	
***V. vermiformis* 18S rDNA gene specific qPCR primers and probe [3]**		
Forward Primer	TAACGATTGGAGGGCAAGTC	Stage I: one cycle of 95°C for 5 min<br>Stage II: 45 cycles of 95°C for 20 s and 60°C for 60 s
Reverse Primer	ACGCCTGCTTTGAACACTCT	
Probe	6 FAM–TGGGGAATCAACCGCTAGGA–Iowa Black FQ	
***Legionella* 16S rDNA gene specific fluorescence *in situ* hybridisation probe [4]**		
LEG705	Alexa Fluor 488-CTGGTGTTCCTTCCGATC	Hybridization: 55 ± 1°C for 100 min
**Eubacterial 16S rDNA gene specific fluorescence *in situ* hybridisation probe [5]**		
EUB338	Alexa Fluor 546-GCTGCCTCCCGTAGGAGT	Hybridization: 55 ± 1°C for 100 min
**Eukaryotic 18S rDNA gene specific fluorescence *in situ* hybridisation probe [6]**		
EUK1209	Alexa Fluor 647-GGGCATCACAGACCTG	Hybridization: 55 ± 1°C for 100 min

References:

1. International Organization for Standardization, *ISO/TS12869:2019 Water quality - Detection and quantification of Legionella spp. and/or Legionella pneumophila by concentration and genic amplification by quantitative polymerase chain reaction (qPCR)*. 2019.
2. Qvarnstrom, Y., et al., *Multiplex real-time PCR assay for simultaneous detection of Acanthamoeba spp., Balamuthia mandrillaris, and Naegleria fowleri.* J Clin Microbiol, 2006. **44**(10): p. 3589-3595.
3. Scheikl, U., et al., *Free-living amoebae and their associated bacteria in Austrian cooling towers: A 1-year routine screening.* Parasitol Res, 2016. **115**(9): p. 3365-3374.
4. Manz, W., et al., *In situ identification of Legionellaceae using 16S rRNA-targeted oligonucleotide probes and confocal laser scanning microscopy.* Microbiology, 1995. **141**(1): p. 29-39.
5. Amann, R.I., et al., *Combination of 16S rRNA-targeted oligonucleotide probes with flow cytometry for analyzing mixed microbial populations.* Appl Environ Microbiol, 1990. **56**(6): p. 1919-1925.
6. Lim, E.L., et al., *Application of rRNA-based probes for observing marine nanoplanktonic protists.* Appl Environ Microbiol, 1993. **59**(5): p. 1647-1655.

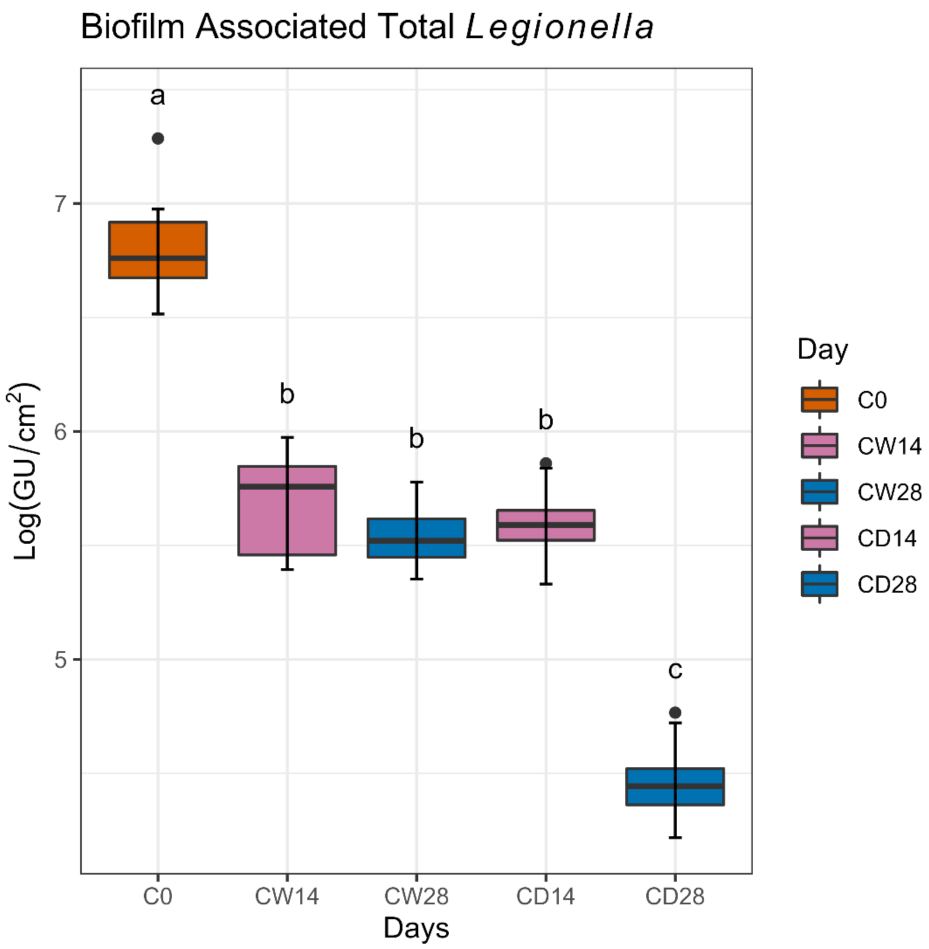

Figure S1: Impact of the flushing frequency on biofilm-associated total *Legionella* quantified by the qPCR assay. The log transformed data are presented as mean ± standard deviation of nine to eighteen replicates. The same alphabetic letter represents statistical similarities at $p < 0.001$ according to Tukey's HSD test. C0: colonization phase, CW14: once-a-week flushing-day 14 sampling, CW28: once-a-week flushing-day 28 sampling, CD14: once-a-day flushing-day 14 sampling, and CD28: once-a-day flushing- day 28 sampling.

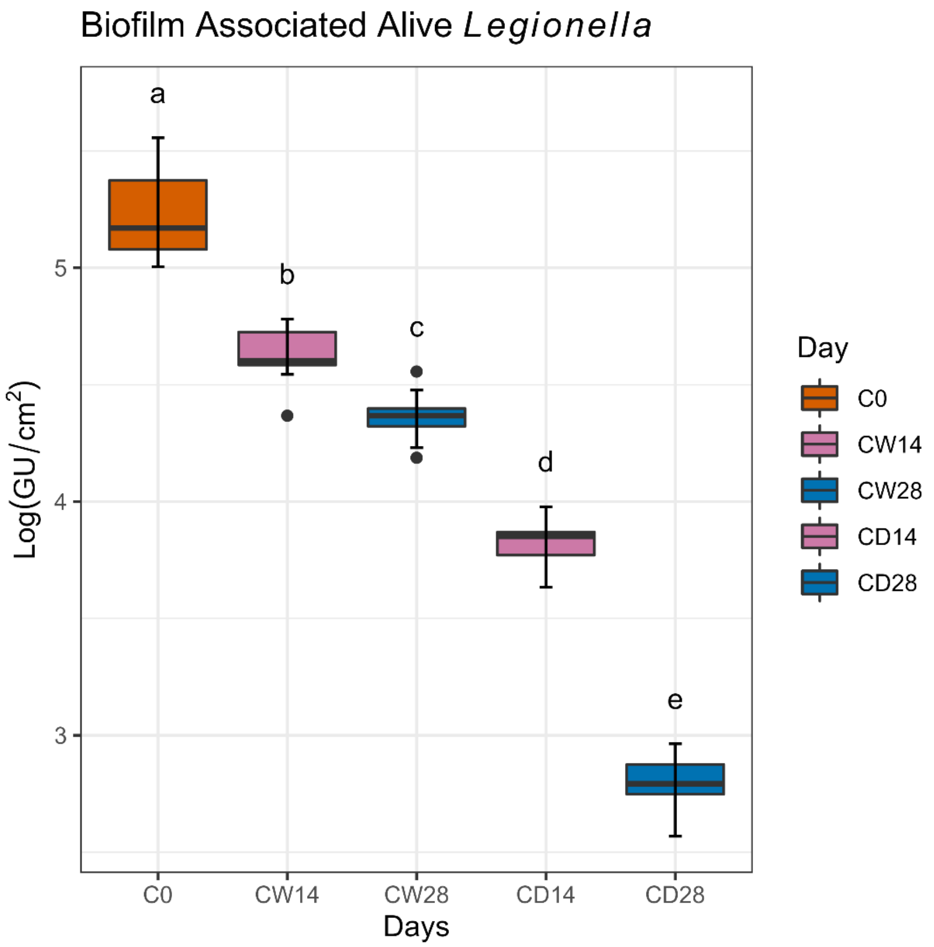

Figure S2: Impact of the flushing frequency on biofilm-associated alive *Legionella* (potentially culturable) quantified by a flow cytometry-cell sorting and subsequent qPCR assay. The log transformed data is presented as mean ± standard deviation of nine to eighteen replicates. The same alphabetic letter represents statistical similarities at $p < 0.001$ according to Tukey's HSD test. C0: colonization phase, CW14: once-a-week flushing-day 14 sampling, CW28: once-a-week flushing-day 28 sampling, CD14: once-a-day flushing- day 14 sampling, and CD28: once-a-day flushing- day 28 sampling.

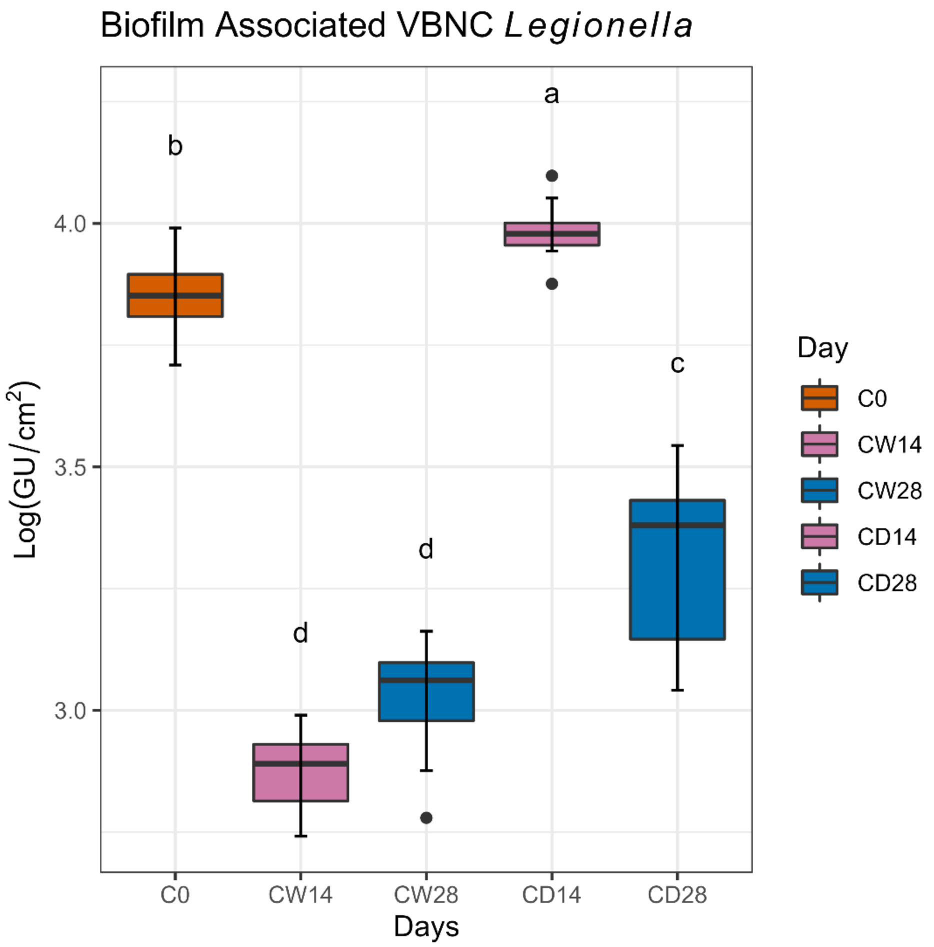

Figure S3: Impact of the flushing frequency on biofilm-associated VBNC *Legionella* quantified by "flow cytometry-cell sorting and qPCR assay". The log transformed data is presented as mean ± standard deviation of nine to eighteen replicates. The same alphabetic letter represents statistical similarities at $p < 0.001$ according to Tukey's HSD test. C0: colonization phase, CW14: once-a-week flushing-day 14 sampling, CW28: once-a-week flushing-day 28 sampling, CD14: once-a-day flushing- day 14 sampling, and CD28: once-a-day flushing- day 28 sampling.

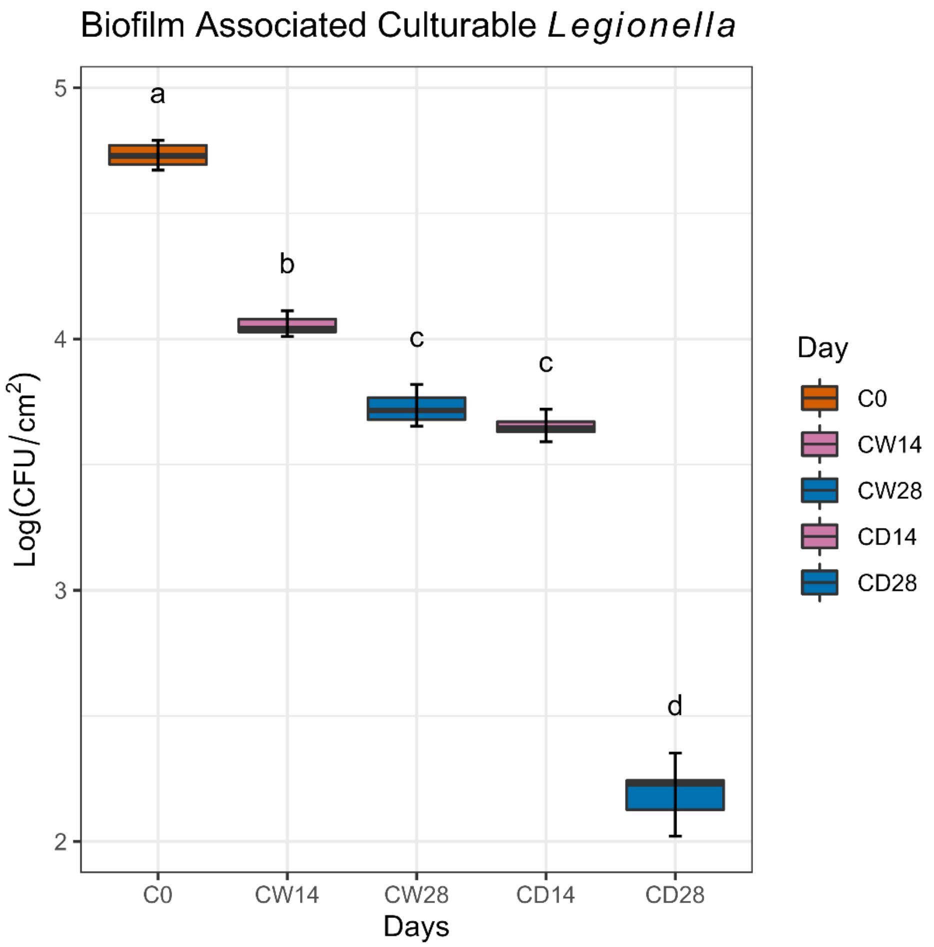

Figure S4: Impact of the flushing frequency on biofilm-associated culturable *Legionella* quantified by "standard culturing assay". The log transformed data is presented as mean ± standard deviation of six to twelve replicates. The same alphabetic letter represents statistical similarities at $p < 0.001$ according to Tukey's HSD test. C0: colonization phase, CW14: once-a-week flushing-day 14 sampling, CW28: once-a-week flushing-day 28 sampling, CD14: once-a-day flushing- day 14 sampling, and CD28: once-a-day flushing- day 28 sampling.

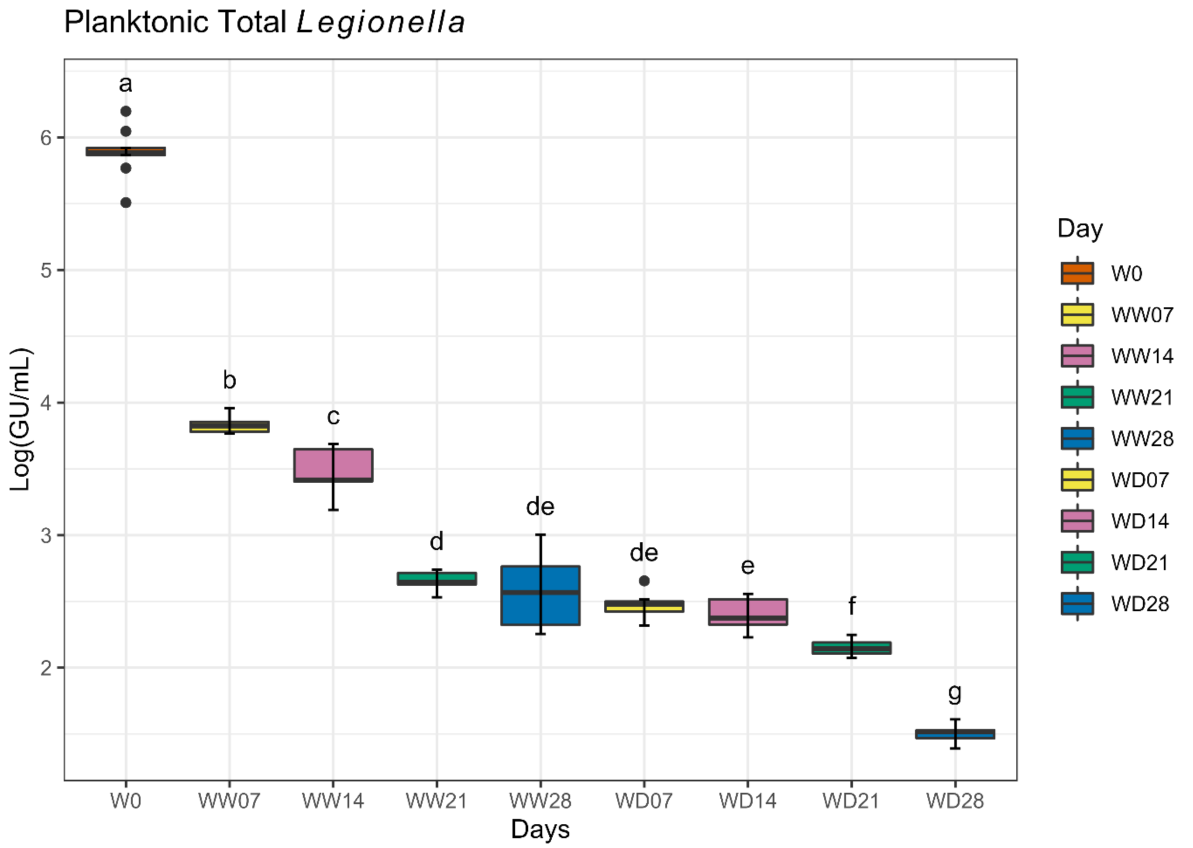

Figure S5: Impact of the flushing frequency on planktonic total *Legionella* quantified by "qPCR assay". The log transformed data is presented as mean ± standard deviation of nine replicates. The same alphabetic letter represents statistical similarities at $p < 0.001$ according to Tukey's HSD test. W0: colonization phase, WW07: once-a-week flushing-day 07 sampling, WW14: once-a-week flushing-day 14 sampling, WW21: once-a-week flushing-day 21 sampling, WW28: once-a-week flushing-day 28 sampling, WD07: once-a-day flushing- day 07 sampling, WD14: once-a-day flushing- day 14 sampling, WD21: once-a-day flushing- day 21 sampling, and WD28: once-a-day flushing- day 28 sampling.

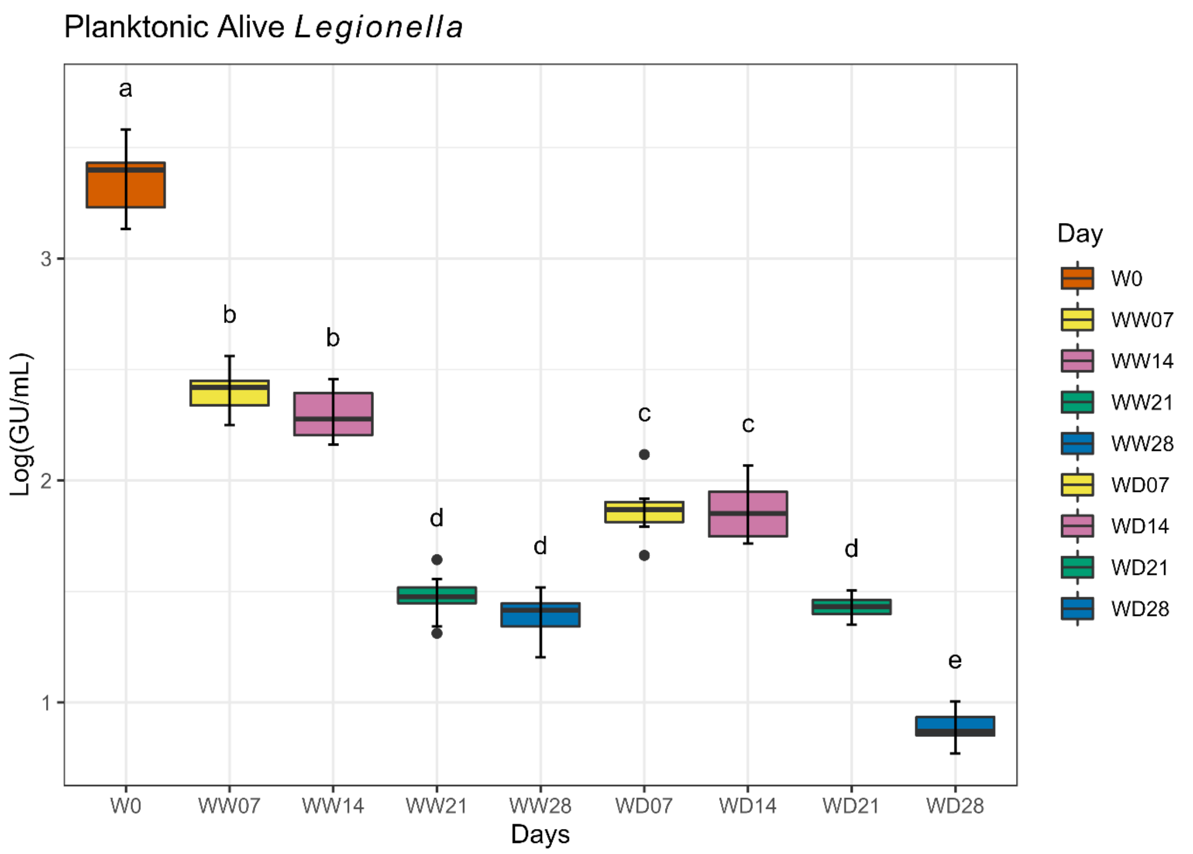

Figure S6: Impact of the flushing frequency on planktonic alive *Legionella* (potentially culturable) quantified by "flow cytometry-cell sorting and qPCR assay". The log transformed data is presented as mean ± standard deviation of nine replicates. The same alphabetic letter represents statistical similarities at $p < 0.001$ according to Tukey's HSD test. W0: colonization phase, WW07: once-a-week flushing-day 07 sampling, WW14: once-a-week flushing-day 14 sampling, WW21: once-a-week flushing-day 21 sampling, WW28: once-a-week flushing-day 28 sampling, WD07: once-a-day flushing- day 07 sampling, WD14: once-a-day flushing- day 14 sampling, WD21: once-a-day flushing- day 21 sampling, and WD28: once-a-day flushing-day 28 sampling.

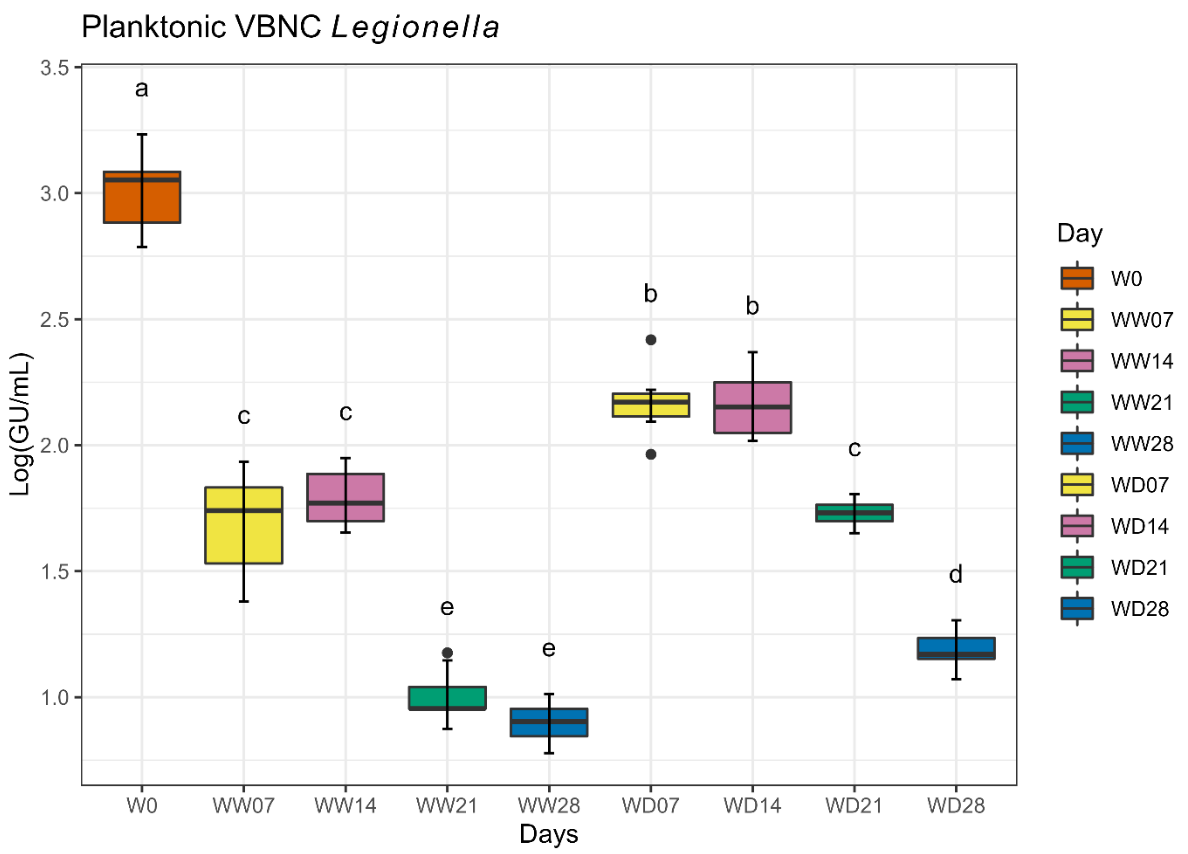

Figure S7: Impact of the flushing frequency on planktonic VBNC *Legionella* quantified by "flow cytometry-cell sorting and qPCR assay". The log transformed data is presented as mean ± standard deviation of nine replicates. The same alphabetic letter represents statistical similarities at $p < 0.001$ according to Tukey's HSD test. W0: colonization phase, WW07: once-a-week flushing-day 07 sampling, WW14: once-a-week flushing-day 14 sampling, WW21: once-a-week flushing-day 21 sampling, WW28: once-a-week flushing-day 28 sampling, WD07: once-a-day flushing- day 07 sampling, WD14: once-a-day flushing- day 14 sampling, WD21: once-a-day flushing- day 21 sampling, and WD28: once-a-day flushing- day 28 sampling.

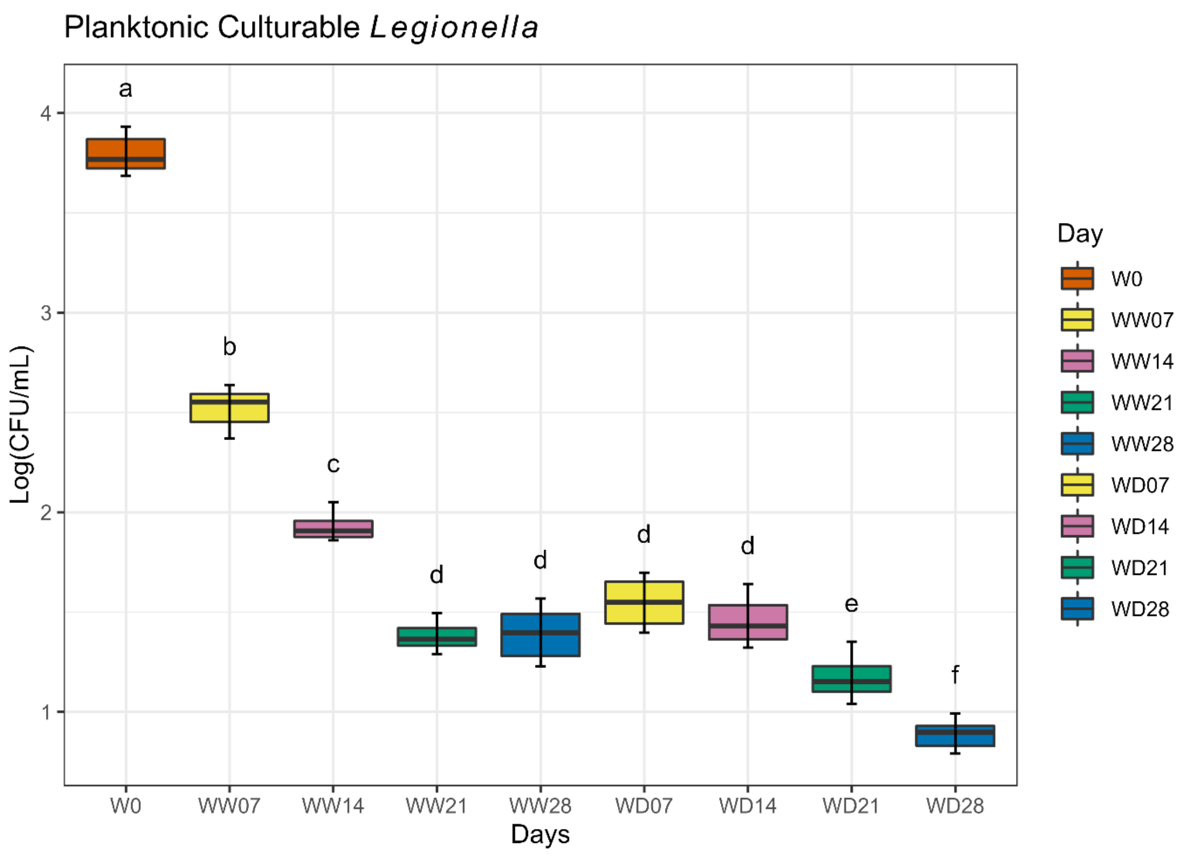

Figure S8: Impact of the flushing frequency on planktonic culturable *Legionella* quantified by "standard culturing assay". The log transformed data is presented as mean ± standard deviation of six replicates. The same alphabetic letter represents statistical similarities at $p < 0.001$ according to Tukey's HSD test. W0: colonization phase, WW07: once-a-week flushing-day 07 sampling, WW14: once-a-week flushing-day 14 sampling, WW21: once-a-week flushing-day 21 sampling, WW28: once-a-week flushing-day 28 sampling, WD07: once-a-day flushing- day 07 sampling, WD14: once-a-day flushing- day 14 sampling, WD21: once-a-day flushing- day 21 sampling, and WD28: once-a-day flushing- day 28 sampling.

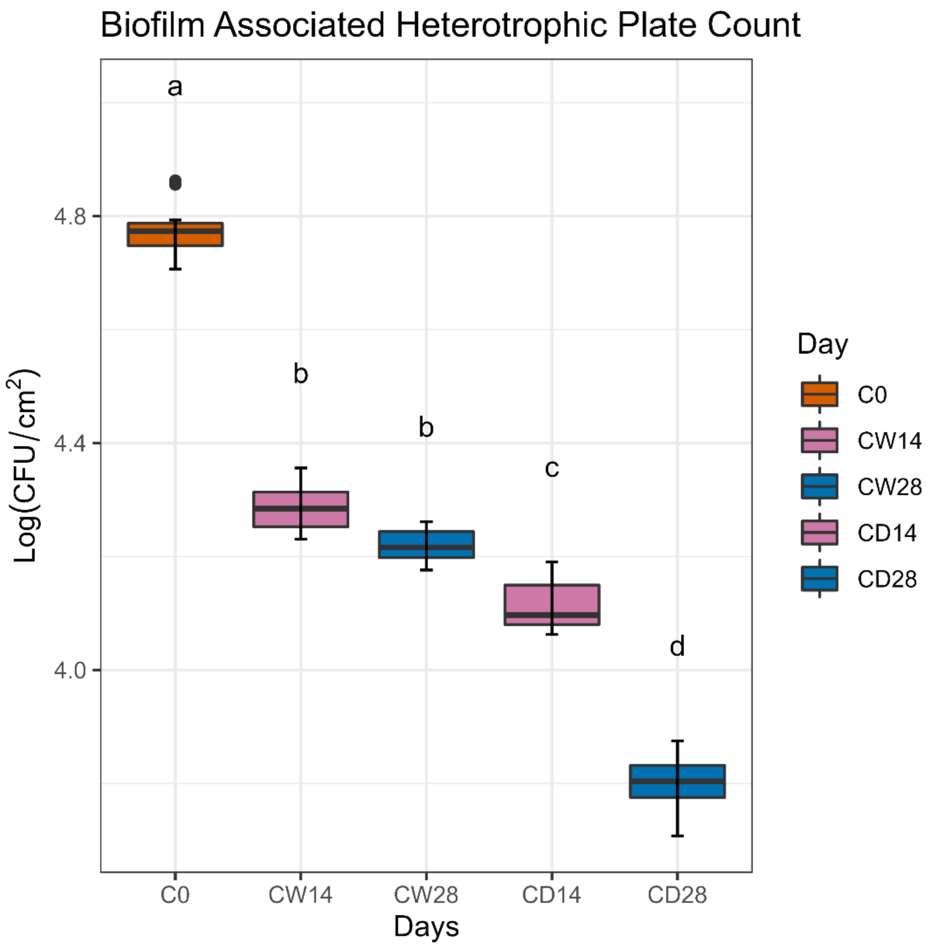

Figure S9: Impact of the flushing frequency on biofilm-associated culturable heterotrophic bacteria quantified by "culturing assay". The log transformed data is presented as mean ± standard deviation of six to twelve replicates. The same alphabetic letter represents statistical similarities at $p < 0.001$ according to Tukey's HSD test. C0: colonization phase, CW14: once-a-week flushing-day 14 sampling, CW28: once-a-week flushing-day 28 sampling, CD14: once-a-day flushing- day 14 sampling, and CD28: once-a-day flushing- day 28 sampling.

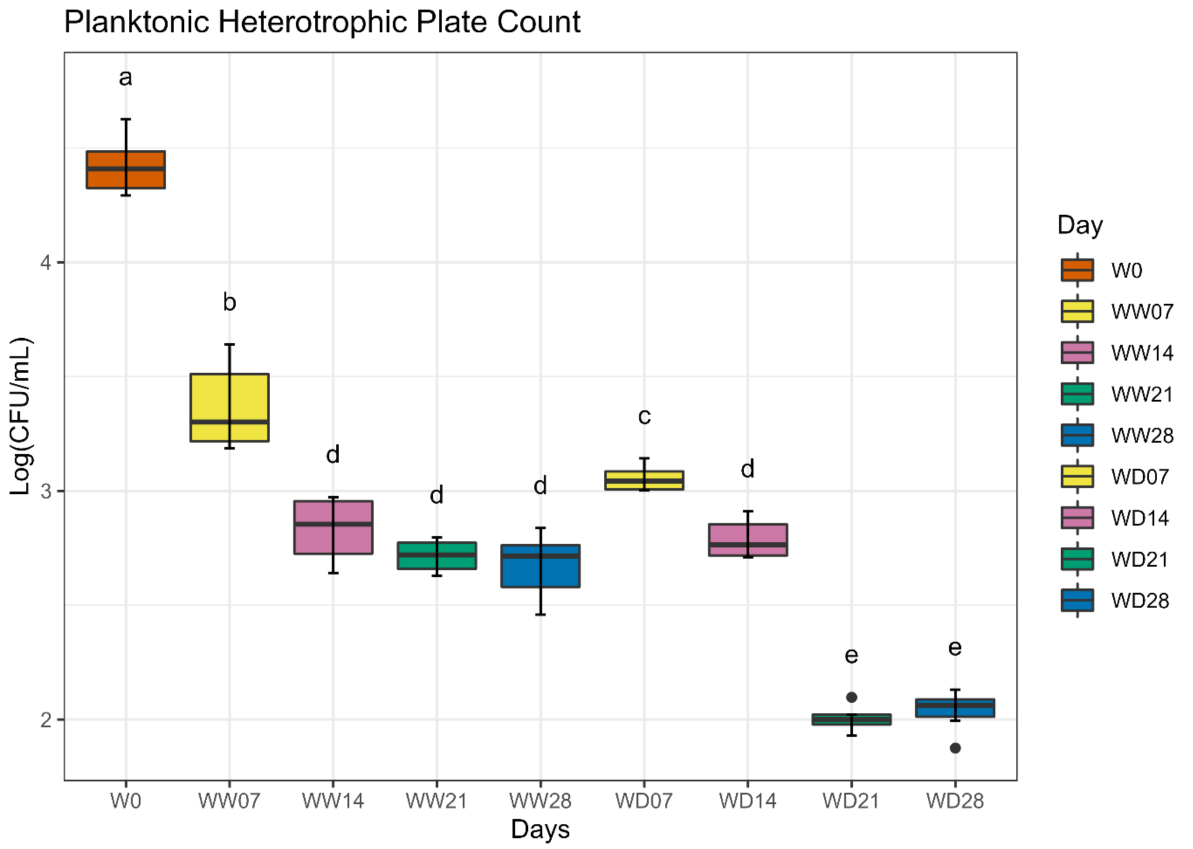

Figure S10: Impact of the flushing frequency on planktonic culturable heterotrophic bacteria by "culturing assay". The log transformed data is presented as mean ± standard deviation of six replicates. The same alphabetic letter represents statistical similarities at $p < 0.001$ according to Tukey's HSD test. W0: colonization phase, WW07: once-a-week flushing-day 07 sampling, WW14: once-a-week flushing-day 14 sampling, WW21: once-a-week flushing-day 21 sampling, WW28: once-a-week flushing-day 28 sampling, WD07: once-a-day flushing- day 07 sampling, WD14: once-a-day flushing- day 14 sampling, WD21: once-a-day flushing- day 21 sampling, and WD28: once-a-day flushing- day 28 sampling.

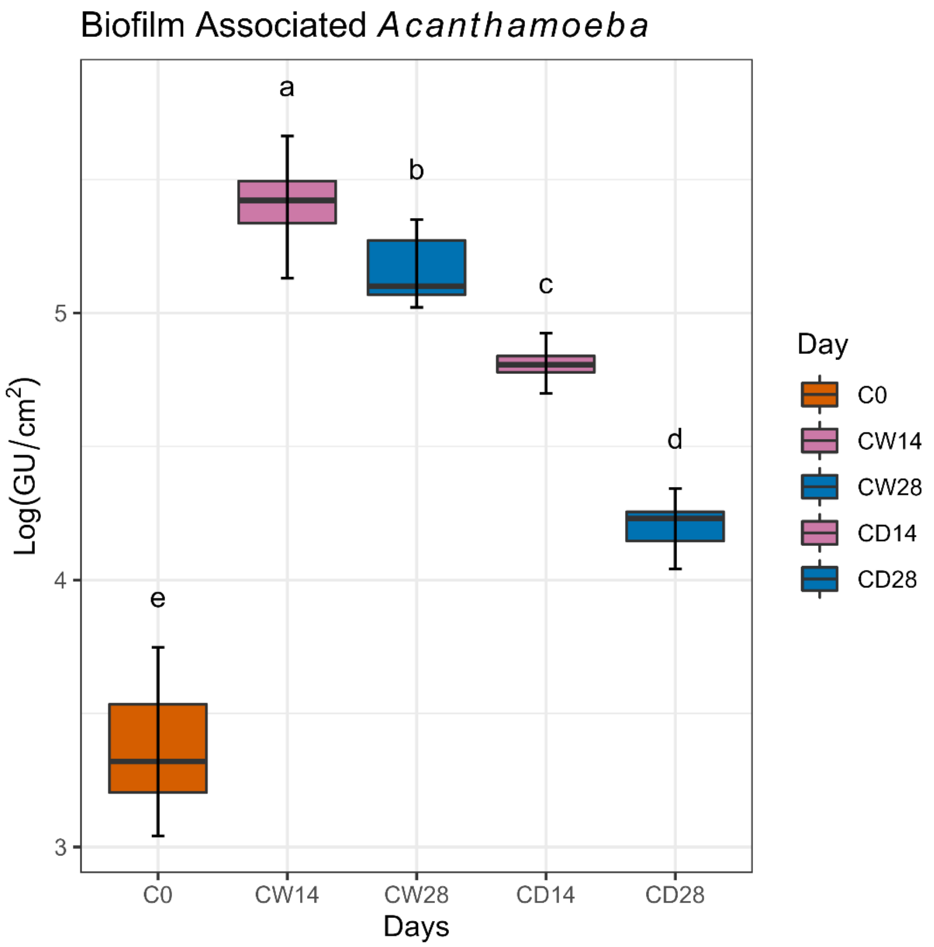

Figure S11: Impact of the flushing frequency on biofilm-associated *Acanthamoeba* quantified by "qPCR assay". The log transformed data is presented as mean ± standard deviation of nine to eighteen replicates. The same alphabetic letter represents statistical similarities at $p < 0.001$ according to Tukey's HSD test. C0: colonization phase, CW14: once-a-week flushing-day 14 sampling, CW28: once-a-week flushing-day 28 sampling, CD14: once-a-day flushing- day 14 sampling, and CD28: once-a-day flushing- day 28 sampling.

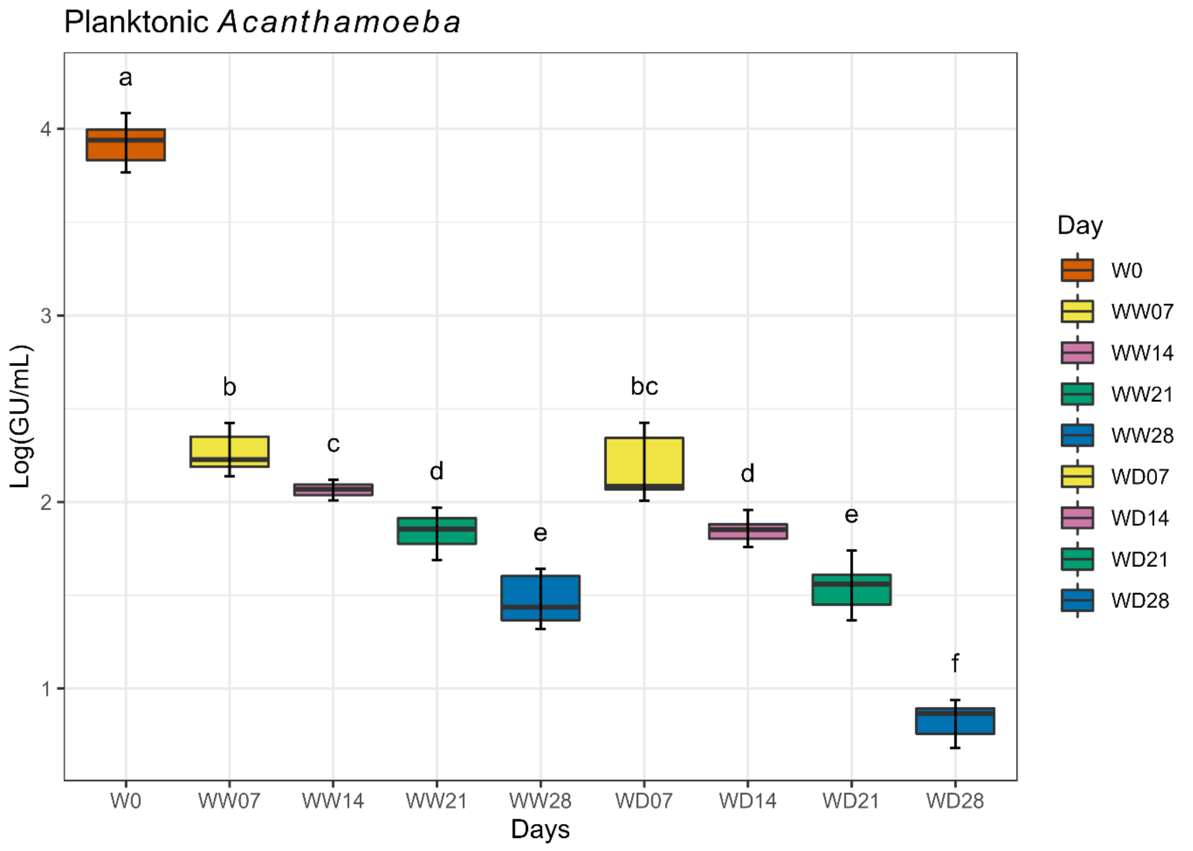

Figure S12: Impact of the flushing frequency on planktonic *Acanthamoeba* quantified by "qPCR assay". The log transformed data is presented as mean ± standard deviation of nine replicates. The same alphabetic letter represents statistical similarities at $p < 0.001$ according to Tukey's HSD test. W0: colonization phase, WW07: once-a-week flushing-day 07 sampling, WW14: once-a-week flushing-day 14 sampling, WW21: once-a-week flushing-day 21 sampling, WW28: once-a-week flushing-day 28 sampling, WD07: once-a-day flushing- day 07 sampling, WD14: once-a-day flushing- day 14 sampling, WD21: once-a-day flushing- day 21 sampling, and WD28: once-a-day flushing- day 28 sampling.

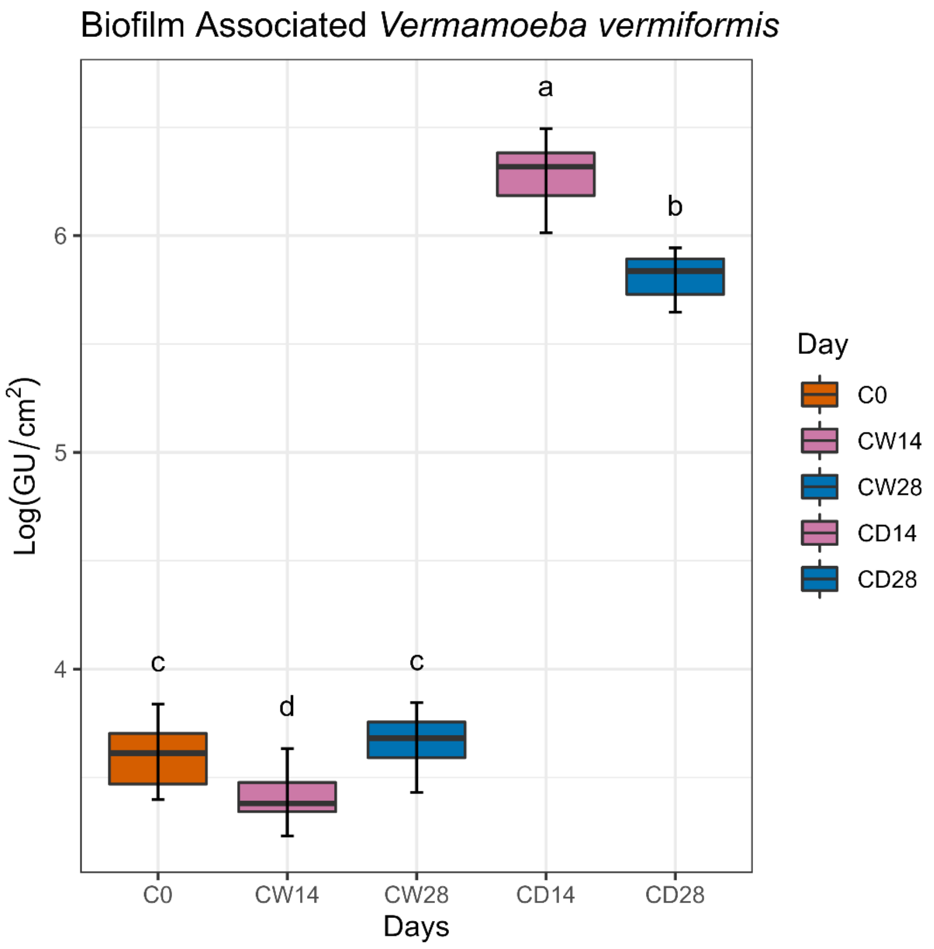

Figure S13: Impact of the flushing frequency on biofilm-associated *Vermamoeba vermiformis* quantified by “qPCR assay”. The log transformed data is presented as mean ± standard deviation of nine to eighteen replicates. The same alphabetic letter represents statistical similarities at $p < 0.001$ according to Tukey's HSD test. C0: colonization phase, CW14: once-a-week flushing-day 14 sampling, CW28: once-a-week flushing-day 28 sampling, CD14: once-a-day flushing- day 14 sampling, and CD28: once-a-day flushing- day 28 sampling.

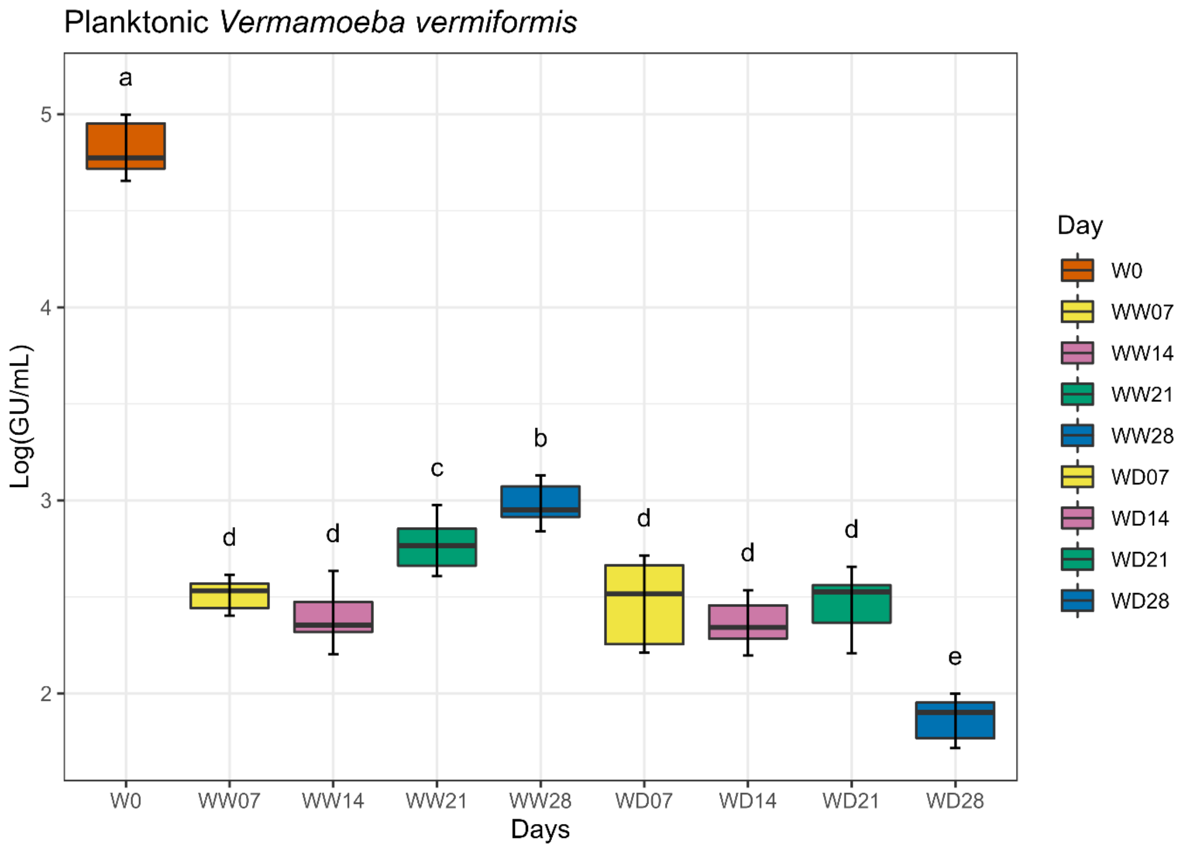

Figure S14: Impact of the flushing frequency on planktonic *Vermamoeba vermiformis* quantified by "qPCR assay". The log transformed data is presented as mean ± standard deviation of nine replicates. The same alphabetic letter represents statistical similarities at $p < 0.001$ according to Tukey's HSD test. W0: colonization phase, WW07: once-a-week flushing-day 07 sampling, WW14: once-a-week flushing-day 14 sampling, WW21: once-a-week flushing-day 21 sampling, WW28: once-a-week flushing-day 28 sampling, WD07: once-a-day flushing- day 07 sampling, WD14: once-a-day flushing- day 14 sampling, WD21: once-a-day flushing- day 21 sampling, and WD28: once-a-day flushing- day 28 sampling.
